# Recent Insights into the Research of (Bio)Active Additives for Advanced Polymer Materials

**DOI:** 10.3390/polym17233139

**Published:** 2025-11-26

**Authors:** Cornelia Vasile, Gladiola Tantaru, Andreea Creteanu

**Affiliations:** 1 Physical Chemistry of Polymers Department, “Petru Poni” Institute of Macromolecular Chemistry of the Romanian Academy, 41A Gr. Ghica Voda Alley, 700487 Iași, Romania; 2 Department of Analytical Chemistry, Faculty of Pharmacy, “Grigore T. Popa” University of Medicine and Pharmacy, 16 University Street, 700115 Iași, Romania; 3 Department of Pharmaceutical Technology, Faculty of Pharmacy, “Grigore T. Popa” University of Medicine and Pharmacy, 16 University Street, 700115 Iași, Romania; andreea.creteanu@umfiasi.ro

**Keywords:** active additives, biological active additives, natural extracts, essential oils, nanoparticles, antimicrobial, antifungal, antioxidant, controlled/targeting release, green extraction, green synthesis, encapsulation

## Abstract

This review is an exhaustive analysis of the recent progress in the research of active and biologically active additives/ingredients. Fast-developing innovations during the last 3–5 years are included in every stage of their preparation from various resources, with valorization of the by-products and waste, characterization, arising problems with their applications, and an important role in the production of the advanced, high-performance materials. The two main well-known classes as natural (bio)active additives and nanoscaled active additives are discussed as it concerns, their types, classification, characteristic mode of action, green extraction and preparation, green synthesis, supplementary processing performed to achieve the suitable stability of the systems by micro-/nanoemulsification/encapsulation, complexation, etc., importance of the composition–activity relationships, biological effects, antimicrobial, antifungal, antioxidant potential, controlled/targeting release of (bio)active agents, the mechanisms of action of antimicrobials, antifungal and antioxidants synthesized in vivo or derived from the human diet, analytical methods for BACs characterization, degradation, toxicity concerns, a.s.o. The combinations of the two kinds of active additives (including even natural additives prepared at the nanosized level) are frequently used in research and scale-up production of new materials as important innovative solutions and challenges in various application fields, especially when their synergism appears, as will be detailed in the second part of this review.

## 1. Introduction

The two significant trends are in current research, industry, and the economy. The “ReShaping Plastics” highlights, as an important necessity in the report issued in April 2022, directed to develop efficient directions to assure the principles of the circular economy, focuses on the three core principles: design out waste and pollution, keep products and materials in use at their highest value, and regenerate natural systems in Europe. This can be achieved by implementing policies that encourage long-lasting product design, promoting reuse and repair over recycling, using new business models like product-as-a-service, and creating solutions for the participation of consumers in all stages. The development of degradable and advanced biomaterials/polymers as a new direction includes active ingredients/additives in their formulations able to control headspace composition, extend the shelf life of polymers, and serve as sensors that detect/indicate the target species, such as the indicators for contaminants of food quality and/or in other fields such as agriculture, pharmaceutics, biomedicine, cosmetics, etc. [1,2,3]. The development of efficient directions to assure the principles of the circular economy helps focus on the three core principles: design out waste and pollution, keep products and materials in use at their highest value, and regenerate natural systems in Europe. This can be achieved by implementing policies that encourage long-lasting product design, promoting reuse and repair over recycling, using new business models like product-as-a-service, and creating solutions for the participation of consumers in all stages.

The first direction is focused on plastics used in large quantities (as in packaging, the automotive industry, construction, etc.), generating huge amounts of waste, having very negative environmental impacts on all ecosystems, and also affecting human health [4], etc. Mechanical and chemical recycling of plastic waste must be improved [5,6], or new solutions to obtain sustainable materials will be developed. Different food preservative techniques like salting, dehydration, freezing, smoking, and irradiation are applied in the food industry during processing and packaging [7].

A particular situation has been encountered during the COVID-19 pandemic period when the various business activities and general consumption have been reduced, leading to a decrease in emissions, also because consumers paid more attention to their personal safety and health. This progress has been more due to the slowdown in economic growth, and its impact was generally positive [8]. Recyclable or alternative materials that do not generate microplastics, are biodegradable, and are impermeable are preferred and show a huge market demand for packaging. There is also a growing interest in the use of such sustainable and natural materials as preferred new solutions in agriculture, delivery systems, and cosmetics. It was estimated in 2020, by the EC, that 56% of consumers are influenced in their purchasing decisions by environmental concerns, and 67% prefer the products that are better for the environment, even if such products are more expensive [9,10].

It could be concluded that new eco-friendly sustainable directions should be developed based on novel natural products and derivatives as main components and also containing selected active agents/additives showing antimicrobial, antifungal, antioxidant, and other desired effects (i.e., accelerating treatments for cancer, diabetes, and other diseases) and nanotechnology to obtain advanced functional materials useful in various application fields such as agriculture, food preservation and packaging, biomedicine, cosmetics, etc. Intensive studies possessing a very rich basis of interest are now included in academic programs and research to find innovative new solutions to develop high-performance materials. The latest release from the AntiBase Library–Wiley Identifier of Natural Products as “A natural product database for compound screening and other applications in natural compounds research” includes more than 105,300 compounds, with the latest release of Natural Product Atlas with over 9500 more compounds [11,12].

To achieve such objectives, important changes must be introduced in the entire cycle of the development of new sustainable materials with suitable properties, high performance, and more; those that assure smart and sensory characteristics, functionality, and biodegradability; and those that are recyclable and also should be obtained by easy, scalable technologies, environmentally friendly procedures at low prices, etc.

Many reviews have been published on certain types of active additives or distinct fields of interest, which have continuously increased, although natural bioactive agents have been considered since ancient times because of their health benefits. Special topics and innovative solutions have been developed to solve problems, creating new materials, such as the extended use of active compounds [13,14,15,16,17,18,19] as eco-friendly, (bio)active compounds to replace the synthetic ones that affect humans and the environment, etc. New types of phytochemicals, plant extracts, essential oils (EO), mushroom extracts, and/or marine products, etc., have been identified [20,21,22,23,24], with different functions as antibacterials, antioxidants [25], preservatives [26], natural pigments, edible coatings and films [27,28,29], smart/intelligent packaging, materials revolutionizing biomedicine by using phytochemicals or nanobiopolymers in human health [30,31], treatment of wounds [32], drug delivery [33], specific natural resources such as peppermint and menthol for pharmacological and clinical applications [34], new cosmetic products [35], and many others.

Natural (bio)active additives together with/or active nanosized additives are new, valuable options to obtain innovative, interesting new materials with large applications in various fields. Both classes of active additives offer an opportunity through the deep, detailed studies and the sustainable circular economy for a smarter, greener future, and, according to the green chemistry principles of development, will be achieved. All aspects related to resources, cultivation area, or synthesis procedures determine, to a large extent, both characteristics of the active additives and, more importantly of the properties of the materials containing them, useful for different applications. Important advances in each domain of preparation/characterization/utilization have been registered in recent decades for both kinds of additives. A continuous bibliographic documentation and the methodology for identification/characterization by deep examination and critical analysis of the bibliographic sources of relevant literature is necessary and pertinent to reveal the increasing role of (bio)active compounds (BACs) as additives/ingredients in the various research fields and daily lives of communities.

The aim of this review is to provide some new insights and innovative biobased/sustainable solutions resulting from the incorporation of the natural (bio)active additives and/or nanosized compounds as active additives in the development of new polymeric materials. The review is based mainly on the 2022–2025 publications (for both types of additives, and especially for nanosized ones), and it updates information useful for selecting topics and materials suitable as active additives both for applications and also for future research on natural and nanosized additives. It should be useful in (i) precise and sustainable agriculture/farming, emphasizing on integration of nanotech solutions in research and practice or the latest breakthroughs as nano-fertilizers and nano-pesticides, with advanced nano-sensors; (ii) impact of nanotechnology in food preservation and packaging, environmental sustainability through nanotechnology innovations to extend food shelf life and safety, also in (iii) biomedicine to treat some diseases, (iv) in cosmetics, a.s.o. One of the recent important research/scale-up directions is based on detailed composition–activities relationships of the BACs, which led to the discovery of new components/compositions/activities [36,37,38]; that is why the review starts with the presentation of the main classes of the natural and nanoscale BAC types/components. As sources of BAC and biopolymers are mentioned, especially by-products, residual materials, and waste from various sectors to be processed and valorized by new efficient and sustainable processes (emulsification, encapsulation, complexation), green syntheses of different nanoparticles (NPs), etc. The recent considerations on the mechanisms of antimicrobial, antifungal, antioxidant activities, controlled/targeting release of (bio)active agents, their detailed characterization by analytical methods, toxicological issues, degradation behavior and recent developments on the applications of the active (bioactive) natural additives/ingredients and active NPs in various sectors are only mentioned in each section (these applications will be the topic of the second part of this review).

## 2. Types of (Bio)Active Additives/Ingredients

**Figure 1:**
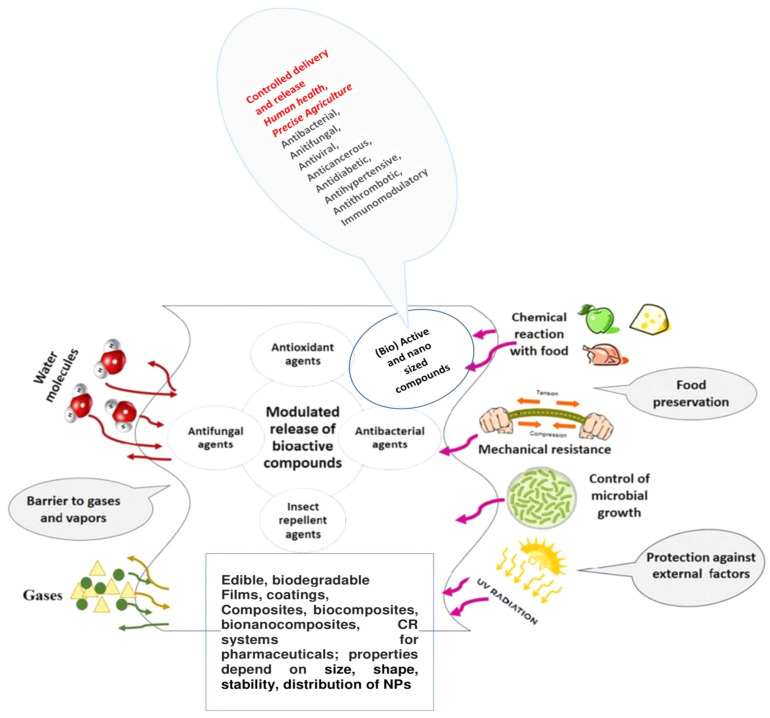
Main effects of the (bio)active compounds on the physico-chemical and biological properties of polymeric materials, Have been written: some applications with red and activities with black. [39,40,41,42,44,46,47,48].

Most types of materials are produced using different additives. An additive is defined in the Federal Food, Drug, and Cosmetic Act as “any substance the intended use of which results or may reasonably be expected to result—directly or indirectly—in it becoming a component or otherwise affecting the characteristics of any food/material”. A wide variety of active agents/additives with various functionalities may be incorporated into the advanced materials to provide the desired activity, such as *preservatives*, *antimicrobials* (e.g., *natural* extracts and essential oils (EOs), antimicrobial peptides, metal–organic frameworks (MOFs), and many more), *antifungals*, *antioxidants*, *insect repellents*, *absorbing*/*scavenging or releasing*/*emitting properties* (e.g., *for oxygen*, *carbon dioxide*, *ethanol*, *ethylene*, *sulfur dioxide*, *odors*/*flavors*, *moisture and liquids*, *and taste*), *pigments*, *colorants*, etc. Table 1 and Figure 1.

**Table 1:** (Bio)active agents used in advanced polymeric materials for agriculture, food preservation and packaging, biomedicine, cosmetics, etc. [39,40,41,42,43,44,45,46,47].

Antimicrobials/Antifungals	Antioxidants/Stabilizers	Oxygen Scavengers	Ethylene or Oxygen Scavengers	Carbon Dioxide Scavengers	Moisture Scavengers	Flavor Emitters	Colorants	Nanoscale Compounds
- Natural extracts - Essential oils and their components (e.g., basil, thyme, oregano, cinnamon, clove, rosemary) - Enzymes - Lysozyme and lactoferrin - Bacteriocins from microbial sources (nisin and natamycin) - Naturally occurring polymers as *polysaccharides* (chitosan, alginate, carrageenan, and their derivatives), *M. charantia* bioactive polysaccharides (MCPs) - *Antimicrobial peptides* (pAMPs) - Metals (Ag, Au) - Metallic oxides (TiO_2_, ZnO) - Organic acids (e.g., sorbic, propionic, and citric acid) - Sodium benzoate, etc., All approved to be used in contact with food	- Natural extracts as hemp extract rich in cannabidiol and cannabis chromene - Essential oils and their components - Phenolic compounds - α-tocopherol - Carvacrol - Quercetin - Catechin - Thymol - Pectin - Lignin - Silver - Copper - TiO_2_ - ZnO	- Curcumin - Laccase - Glucose oxidase - Gallic acid - Ascorbic acid - Fe - Pd	- MOFs - Zeolites - Halloysite - Activated carbon - Metal oxides - TiO_2_ - KMnO_4_ - Nanotubes (HNTs)	***Emitters*** - Sodium bicarbonate - Citric Acid - Ferrous carbonate *Scavengers* - Ca(OH)_2_ - Na_2_CO_3_, - Mg(OH)_2_	- Fructose, - Cellulose, - NaCl, - Ca(OH)_2_, - Zeolites - Montmorillonite - Halloysite nanotubes	- Some EOs - Vanillin - -Allyl isothiocyanate - Citral - Ethyl hexanoate	- *From natural sources* - Mineral ores, - Plants, -Insects, and -Animals - Synthetic dyes - Microbial pigments	*Inorganic NPs* - Ag NPs - Au NPs - ZnO NPs - -TiO_2_ NPs - Cu and CuO NPs - Fe and Fe oxides NPs - Silica - Carbon nanotubes - Graphene nanosheets - Nanoclays: montmorillonite (MMT) NPs, kaolinite, and silicate nanoplatelets *Biopolymer NPs* - Starch nanocrystals - Cellulose nanofibers and nanowhiskers - Chitosan and - Chitin whiskers and nanofibers (electrically conducting - Alginate NPs - Lignin NPs - Protein NPs (collagen, gelatin, β-casein, zein, and albumin)- Nanofibres - Nanotubes (hollow nanofiber) and nanorods)

*Antimicrobial* agents *kill or destroy* microorganisms or *inhibit* the growth of microbes (as (micro) bacteriostatic agents); they may be antibacterial, which work against bacteria; antifungal, against fungi; or microbiocidal, which kill microbes or inhibit their growth—*inhibitors*. *Antioxidants*, as a group of preservatives, prevent fats, oils, and the foods containing them from becoming rancid or developing an off-flavor, protect fresh fruits such as apples from turning brown when exposed to air, etc. *Preservatives* prevent decomposition by microbial growth or by undesirable chemical changes in various products such as food, beverages, pharmaceuticals, drugs, paints, biological samples, cosmetics, wood, and many other products. *Natural pigments* having enhanced stability are explored as sensors of food properties in intelligent/smart packaging [17,39].

The BAC addition to various products/materials improves their quality (*antibrowning agents*), extends shelf life (antioxidants and antimicrobials), enhances sensory properties (flavor, color, and texture), and adds health benefits. *Nutritional constituents* considered to have a therapeutic or health benefit are nutraceuticals (prebiotics, probiotics, vitamins, and minerals) or bioceuticals and nanonutraceuticals as micronutrients. Active additives can also act as *functional compounds*, *biologically active agents*, *and drugs for various diseases*; *barriers to gases and vapors*; *preservatives*; *and protection against external factors such as UV radiation and chemical reactions with food*; *or exhibit multiple effects*, especially when some of their mixtures are used. Selected additives depend on the application needs [40].

As different types of ingredients/additives exist, a special place is occupied by those that are Generally Recognized as Safe (GRAS) by both the EC and the U.S. Food and Drug Administration (FDA), such as thyme EO, ZnO as a food additive, and MgO NPs, an interesting inorganic material [41]. “Food active ingredients” aim to extend shelf life and increase the nutritional value of the packaged product [42,43], and in many advanced materials for food packaging, medical devices, cosmetics, electronics, textiles, etc., they are used to induce desired activities like antimicrobial, antifungal, and antioxidant activities and controlled delivery and release of active compounds.

A bioactive compound (BC) has a biological activity related to its ability to modulate one or more metabolic processes, promoting better health conditions, and as a food component, it has an effect on the whole body or on specific tissues or cells. BCs are distinct from nutrients because they are not essential, and, currently, there are no recommended daily intake values. Bioactive natural compounds present large differences in their chemical structures (hydrophilic or lipophilic) and distribution in nature (specific to vegetable species or ubiquitous) and are found in fruits, vegetables, and whole grains in a wide concentration range; they exist both in foods and in the human body. All these explain their possible variable site of action, the differences in bioaccessibility, bioavailability, bioactivity, prebiotic effects, and their effectiveness against oxidative species, and the specificity of the biological action [44].

It is very difficult to achieve simultaneous improvement in multiple structural functions by using conventional structural materials. A multifunctional material that performs multiple functions in a system due to its specific properties is preferred, as it is a multifunctional packaging that delays food decay by retarding the ripening, dehydration, microbial invasion, etc.

*Microbial natural pigments* offer a sustainable and economically viable alternative to synthetic colorants; they are relatively non-toxic and exhibit significant health benefits; therefore, the growing demand for such natural products appears. *Emulsifiers*, *stabilizers*, *and thickeners* offer the texture and consistency to foods that consumers expect.

Food undergoes spoilage due to microbial contamination, enzymatic degradation, oxidation, excess ethylene, uncontrolled temperature, undesirable moisture, and inadequate pH. The oxidation process in food occurs by the generation of free radicals that accelerate enzymatic browning and microbial proliferation, leading to food degradation [45]. Such processes can modify the characteristics of the food, including color, flavor, aroma, texture, and nutritional value, making the food unfit for consumption. Therefore, a food additive should assure at least three cumulative requirements, such as *preservative*, *coloring*, and *anti-caking* effects, as it has been defined in Regulation (EC) No 1333/2008. Preservatives such as *synthetic or natural antioxidant compounds* have been incorporated into active packaging systems to prolong shelf life [49]. Biopolymers with active biomolecules as internal coatings or external layers in biocomposites preserve the product freshness while upholding structural integrity and fortifying barrier properties, thus shielding bread from moisture, oxygen, and external influences [14,15,18,50,51].

Synthetic antioxidants/preservatives such as butylated hydroxytoluene (BHT), butylated hydroxyanisole (BHA), or tert-butyl hydroquinone (TBHQ), nitrates/nitrites, sulfites, sodium benzoate, propyl gallate, and potassium sorbate are still used in the food industry, but they show potential risks to human health (headaches, allergies, and cancer) [52,53]. By replacing or reducing synthetic additives with the natural ones described here, the preservation and microbiological safety of foods is improved, contributing to the sustainability of the food industry.

Active compounds useful in foods, nutraceuticals, pharmaceuticals, etc., include extremely heterogeneous classes of compounds such as polyphenols, carotenoids, tocopherols, phytosterols, organo-sulfur compounds, vitamins, omega-3 fatty acids, organic acids, nucleosides and nucleotides, and microelements; therefore, they showed a gaining interest also due to health benefits, which can help to reduce the risk of certain chronic diseases [53,54]. Some biopolymers also act as active additives, mainly when they are at the nanosize level (Table 1: starch, cellulose, lignin, chitosan, chitin, alginates, gelan, etc.) [55,56,57].

### 2.1. Natural Active Additives

The three main categories of natural active additives are phytochemicals, natural extracts, and essential oils. Phytochemicals are a broad category of chemical compounds found in plants, while natural extracts and essential oils are specific types of phytochemicals as complex mixtures of compounds extracted from plants using particular procedures. The main difference between essential oils and natural extracts is the obtaining process. While both are extracted from different parts of the plant, the processes are very different. Essential oils need to be extracted through distillation or steam distillation (commercially, it is the most frequently used method), while extracts are soaked in a liquid to isolate the flavor, so they are diluted with respect to EOs. Natural extracts can be used in higher concentrations ranging from 3 to 20%. Phytochemicals contribute to color, smell, and taste and provide health benefits, while essential oils are primarily used for their strong aromas, flavors, and potent biological activities, such as antioxidant, anti-inflammatory, and antimicrobial effects.

#### 2.1.1. Phytochemicals

Phytochemicals from medicinal plants and herbs (derived from various sources such as herb shoots, flowers and leaves, grains, seeds, fruits, vegetables, legumes, nuts, etc.) are produced for the protection of their physiological functions. Plant-based bioactives (biologically active compounds) (more than a thousand), possessing antioxidant and antimicrobial properties, show a significant impact on human health [31]. They have a dosage-dependent effect on human health, demonstrated by in vitro and in vivo studies. Phyto-nano-crystalline chemicals derived from various botanical sources have an increasing potential as preservatives in foods labeled as GRAS, including essential oils, such as those isolated from plants (such as clove EO from *Syzygium aromaticum*, basil EO from *Ocimum basilicum*, black pepper EO from *Piper nigrum*, cannabis from *Cannabis sativa*, citronella EO from *Cymbopogon nardus*, and rosemary EO from *Salvia rosmarinus*), cinnamon, oregano, thyme, ginger, lavender, eucalyptus (*Eucalyptus globulus*) [22,58], pomegranate by-products [59], etc. They are listed in the natural additives/preservatives authorized feed additives list for the European market [60].

The bioactive compounds extracted from plants are very good as *antimicrobial agents*, inhibiting the harmful activity of bacteria, fungi, and viruses, and are also very interesting not only for their biological activities and valuable therapeutic properties but also for their biocompatibility, renewability, biodegradability, etc.

Antioxidant activity promotes cellular health by lowering oxidative stress responsible for aging and the onset of chronic diseases. A diet rich in phytochemicals determines a lower incidence of lipid peroxidation, cardiovascular disorders, cancer, and other illnesses. The β-glucans, as active compounds of *Echinacea*, stimulate immune responses, enhancing the body’s ability to defend against infections and diseases.

Bioactive additives/agents’ role is imparted by their composition [61,62]. They are selected according to the proposed application after a detailed investigation of their composition and activities. As an example, total phenolic content in fruits of *P. fraternus* ranged from 26.69 to 61.48 mg GAE/gfw (milligrams of Gallic Acid Equivalent per gram of fresh weight) and from 8.89 to 24.69 mg GAE/g fw in leaves of *S. nigrum* plants, and thus, these plant parts are useful for pharmaceutical purposes and health benefits [63]. The biochemical diversity of plant-derived chemicals determines their antimicrobial, antioxidant, etc., activities and selected potential applications in various fields of human life, including agriculture, food and nutrition, nutraceutical industries, pharmaceuticals, cosmetics, etc. [21,31,64].

As *phytochemical compounds*, the main sub-classes are carotenoids, polyphenols, isoprenoids, phytosterols, saponins, terpenes and terpenoids, flavones, flavonoids, flavonols, flavanones, isoflavonoids, anthocyanidins, phenolic acids, the stilbenes and the lignans, phytoestrogens, limonoids, alkaloids, glucosinolates and cyanogenic glucosides (e.g., isothiocyanate, phenylethylisocyanate, and idoles), polyacetylenes, lectins, tannins, phytosterols and phytostanols, dietary fibers, and specific polysaccharides. The main bioactive constituents of the phytochemicals are vitamins, peptides, enzymes, bactericides, unsaturated fatty acids, minerals, etc. Detailed classification and sources of phytochemicals are given in [31]. The main components of the phytochemicals and their concentration depend on the source, conditions of the plant cultivation, such as climate and soil, and the extraction method. Because of this wide variety of compounds and compositions, they differ significantly in their bioavailability, structure, and biochemical properties. Medicinal plants contain several phytochemicals and possess antimicrobial/antifungal, antiviral, antidiarrheal, anthelmintic, antiallergic, and antioxidant properties. They could also behave as targeting specific receptors, interrupting disease pathways, and disrupting pathogenic life cycles. Many diverse activities explain their significant interest as potential therapeutic agents with low toxicity and adverse effects in comparison with commonly used chemotherapeutic or radiotherapeutic agents. Bioactive pigments (including chlorophylls, betalains, carotenoids, phycocyanin, and anthocyanins) also show significant antimicrobial, antioxidant, and immunologic properties, being of great interest in the pharmaceutical, food, and other sectors.

The sensory properties of plants, such as taste, aroma, color, and flavor, have been considered useful from ancient times [65,66,67]. During the plant’s metabolism, oxygen is released into the environment, decreasing pollution. Phytochemicals are also powerful nutrients consumed in the form of whole fruits, purees, vegetables, prepacked fruits, and/or vegetable products and supplements, and are associated with health benefits. Many phytopharmaceuticals have pharmacological activities as they can maintain the sound human body functions. As an example, cannabidiol in industrial hemp is a promising functional food ingredient with multivarious health benefits, including anticancer activity, antioxidant activity, anti-inflammatory properties, and anxiolytic effects [68]. Another interesting active additive is peppermint. It contains three main groups of phytochemical constituents, including mainly menthol, flavonoids (such as hesperidin, eriodictyol, naringenin, quercetin, myricetin, and kaempferol), and nonflavonoids as phenol carboxylic acids and it exhibits antimicrobial, antioxidant, anti-inflammatory, immunomodulatory, anticancer, antiaging, and analgesic properties and may be effective in treating various disorders, including gastrointestinal disorders (e.g., irritable bowel syndrome, dyspepsia, constipation, functional gastrointestinal disorders, nausea/vomiting, and gallbladder stones). In addition, peppermint has therapeutic benefits for psychological and cognitive health, dental health, urinary retention, and skin and wound healing, as well as anti-depressant and anti-anxiety effects, and it may also improve memory [34].

It is a continuous research interest to identify novel bioactive chemicals from medicinal plants that could provide creative and easy methods to combat pathogenic microbes due to the rising prevalence of drug-resistant infections and deliver AC in a controlled manner [35,69,70].

#### 2.1.2. Natural Extracts

Active extracts resulting from animals and plants having good antioxidant and antibacterial properties are beneficial for human health, being ideal substitutes for synthetic additives. Plant extracts are produced from various sources, such as fruits and vegetables, herbs and spices, and flowers [20,71,72,73,74,75,76,77]. They are used as active additives in the food and beverage industry, pharmaceutical industries, cosmetics, skincare preparations, etc., because they extend the shelf life of materials, maintaining their original mechanical, optical and thermal properties and imparting new functions, and are very efficient in scavenging free radicals [71,72], exhibiting antioxidant and anti-inflammatory activities depending on the type of extract and its concentration (as crude extracts of *Codiaeum variegatum* stem) [73]. Bacteria, yeast, and molds survive during harvesting, slaughtering, processing, and packaging, and also under adverse conditions applied for food preservation, such as low temperature, modified atmosphere packaging, vacuum packaging, and conventional pasteurization. Environmentally friendly methods are developed for the treatment of some human diseases using the crude extracts of different parts of medical plants [78]. The antifungal effect of leaf extracts from yarrow (*Achillea millefolium* L.), tansy (*Tanacetum vulgare* L.), sage (*Salvia officinalis* L.), and wormwood (*Artemisia absinthium* L.) on fungi of the genus *Fusarium*, major cereal pathogenic fungi such as *Fusarium avenaceum*, *F. culmorum*, *F. graminearum*, and *F. sporotrichioides*, has been evaluated at a laboratory scale [79]. Plant extracts from sage have the highest polyphenol contents (81.95 mg/mL) and flavonoids (21.12 mg/mL), and consequently the highest antioxidant activity compared to other plant extracts. The extracts of sage and tansy plants at a concentration of 20% exhibited a strong inhibitory effect against the tested fungi. Several crude extracts (such as cinnamon, garlic, basil, curry, ginger, sage, mustard, etc.) exhibit antimicrobial properties against both Gram-positive and Gram-negative bacteria. Organic solvents such as ethanol, methanol, acetone, and diethyl ether were used to extract turmeric. It has been found that the 75% ethanol extract of is extremely active against *Aspergillus flavus* growth, while the highest aflatoxin B1 production inhibition ratio was achieved using the 25% ethanol turmeric extract [80].

Biodegradable packaging materials with active antioxidant capability were fabricated based on polyvinyl alcohol–corn starch integrated with beetroot stem waste extract as a packaging material for oxygen-sensitive food products [81] or based on cellulose acetate/potato peel starch as an alternative to petroleum plastics and with good biodegradability [82].

*Sacha inchi* oil, with a high content of linolenic and linoleic acid, acts as a moisturizing agent of lipid source and as a preservative; also, it increases nutritional value when added to food. Physico-chemical qualities of oils and extracts useful for food and cosmetics are affected by cultivation area, seed chemical profile, extraction method, and working conditions (temperature, pressure, drying, time, etc.) [83]. Dual synergistic-assisted extraction presented a higher content of bioactive compounds and antioxidant activities compared with single-assisted extraction. The Cam rice bran could be considered as a source of antioxidant and nutraceutical ingredients [84].

Farhadi and Javanmard [85] developed an active packaging system as fibrous casings based on sugarcane bagasse waste from sugar factories coated with thyme and/or rosemary extract (0–6%). Rosemary and thyme extracts have increased water vapor permeability of the active films and improved sausage lipid oxidative stability. Therefore, fresh fruits and vegetables are vectors for bacteria (such as *Escherichia coli*, *Staphylococcus aureus*, *Listeria monocytogenes*, *Salmonella*, *Bacillus*, etc.), fungi (spoilage yeast, *Aspergillus niger*, *Penicillium*, etc.), and viruses (norovirus, etc.). It has been found that the jujube extract in electrospun PVA nanofiber is useful for strawberry preservation because it has an inhibiting effect on bacteria and fungi (*Bacillus subtilis*, *Escherichia coli*, and *Aspergillus fumigatus*) [86].

The beneficial effect of olive leaf extract on human health is due to its good antioxidant effect being rich in polyphenols, in particular oleuropein, hydroxytyrosol, and tyrosol and it favors the dispersion in different food matrices, such as oils, meat, baked goods, vegetables, and dairy products. It enhances the organoleptic and nutraceutical properties of the products, reducing oxidative damage to fats and proteins [87].

Ethanol extract of propolis exhibits severe antimicrobial activities towards Gram-positive cocci strains belonging to *S. aureus* types. Many pharmaceutical materials with antibacterial, antifungal, antiviral, antiprotozoal, anti-inflammatory, antioxidant, anticancer, and other activities have been prepared using propolis [88].

Natural extracts can replace some antibacterial, antifungal, and antiviral additives, and also they are also currently used as antibiotics.

The global plant extracts market size has been estimated at USD 34.4 billion in 2022, and it is projected to reach USD 61.5 billion by 2027, recording a Compound Annual Growth Rate (CAGR) of 12.3%. The increased demand for plant extracts is explained by the changes in the lifestyle of consumers and their preferences for organic products containing natural ingredients with additional functional properties. Various plant extracts have been incorporated in different types of polymer-based biomaterials, including films, hydrocolloids, sponges, foams, hydrogels, and nanofibers, and these have been widely applied in the biomedical, tissue engineering fields, and wound dressings due to their excellent biocompatibility, antibacterial and anti-inflammatory properties, and low side effects [76].

#### 2.1.3. Essential Oils (EOs)

The EOs are considered the most widely traded products. It is appreciated that 300 EOs have commercial significance, their global production exceeding 70,000 tons annually and potentially exceeding USD 15 billion by 2025 [89], with the global production being dominated by the USA, Brazil, India, and China. Essential oils, such as secondary metabolites of herbs and spices, as liquid extracts are obtained as volatile compounds from various parts of plants and rhizomes. Each EO has its own unique characteristics. Their chemical composition has been determined by coupling different methods (Folin–Ciocâlteu method, GC-MS, and flame ionization detectors, respecting the AFNOR/ISO standard limits), and they have been FDA approved as GRAS for food use [90]. EOs from different plant species contain more than 200 volatile and non-volatile constituents as complex mixtures of numerous volatile substances (20–80), including terpenes (hydrocarbon group), sesquiterpenes, terpenoids, and non-terpene compounds (oxygenated group). All kinds of EOs contain two or three major components (20–70%), which define their characteristics, while the other components are present in trace amounts. The volatile compounds account for >80–90%. Due to their rich chemical and unique composition, especially in specific aromatic compounds, EOs have many interesting properties and often impart a characteristic aroma and taste to foods. They have efficient antimicrobial, antibiofilm, antioxidant, and antifungal properties and could be a solution for novel preventive and/or antibiotic-independent therapeutic measures [91,92,93,94]. EOs have a hydrophobic nature and lower density than water, being lipophilic, and they are soluble in organic solvents. For example, in *Origanum* species, the essential oil contains 30% carvacrol and 27% thymol as major components. Composition of EOs and of many other properties of the products resulting from biomass also depends on many factors, such as the type of the bioresources (crops, wood, vegetal, microbial biomass, etc.), plant organ, age, cycle stages of plant life, soil composition, climate change, geographical area, harvest season, habitat, drying processes of plant raw material, extraction techniques and methodology, storage time and conditions, etc. [95,96]. The main functions of the EOs are antimicrobial and antioxidant activity, immune function, empowerment, and insect repellents. EOs have also been tested to control fungal diseases in cereal grains [97]. These functions determine their use in agriculture as feed additives, the food industry as preservatives, as suitable alternatives to conventional preservatives, the pharmaceutical industry, the cosmetic industry, health care, etc. The biological, biocidal, and antioxidant properties of EOs are related to the presence of phenolic or terpenoid compounds in their composition [37]. Each essential oil has a different biological activity, physico-chemical properties, and aroma; thus, it is important to select the most appropriate one or combination for each specific application or type of food [29]. Their feasible applications are mainly restricted to disposable food wrappers for fast foods that do not require improved water barrier properties. Their use brought an important development in novel intelligent and active packaging because these preserve foods by keeping temperature, moisture level, and microbial and quality control of the food, releasing antioxidant and/or antimicrobial agents, increases in the UV barrier properties, and surface hydrophobicity [28,98,99,100].

Functionalization reactions are sometimes applied to achieve desired high-performance properties, which can be successfully performed by in situ reactive extrusion using natural phenolic compounds such as pyrogallol exhibiting antioxidant and antimicrobial properties [101]. The obtained products are divided into adsorbent and releasing systems (e.g., oxygen scavengers (used in packaging pasta, milk powder, biscuits), ethylene scavengers, liquid and moisture absorbers, taste and odor absorbers or releasers, antimicrobials, etc.). The incorporation of essential oils significantly increases elongation at break and enhances UV barrier properties.

The migration of the active ingredient as EOs (clove oil, grapefruit oil, and tea tree oil) from the films into the acetic acid solution did not exceed 10 mg/kg, which is an acceptable value according to the European Union restrictions. Moreover, the antimicrobial agent is slowly released from the sachet, maintaining the freshness of the packaged food.

*Antimicrobial food packaging* presents a strategic defense against spoilage and pathogens as a solution for the need for sustainable food security and waste reduction. Some of these compounds show stronger antimicrobial effects in the vapor phase than in the liquid phase; this quality makes them good candidates as non-contact preservatives. A solution to exploit the antimicrobial activity of the EOs at reduced doses consists of consideration of the volatile bioactive compounds. Some EOs are particularly suitable for applications such as air disinfection and other related fields [102]. The essential oils’ effects also include those of anticancer, anti-inflammatory, and anti-viral agents; wound healing as a potential therapeutic option for cutaneous wound management; an alternative to conventional antibiotics; use in animal feed; and use as food preservatives, because of their antimicrobial action against many types of bacteria. Tea tree oil with hesperidin-loaded lipid nanoparticles exhibits a superior therapeutic potential option for cutaneous wound management and wound-healing capabilities with sustained release and superior synergistic efficacy [103]. EOs from *M. alternifolia *and *C. nardus* show the greatest fungicidal effect (>90%). The combined use of the different EOs indicated a synergistic effect against *F. graminearum* and *A. flavus* and an antagonistic effect against other isolates. *C. nardus* EO is effective in the control of storage pathogens, and by combining with other EOs, it can improve their antifungal effects [104,105]. As it concerns the antioxidant activity (determined by 2,2-diphenyl-1-picrylhydrazyl (DPPH) and 2,2′-azino-bis-3-ethylbenzthiazoline-6-sulfonic acid (ABTS) methods), it has been established that the clove oil exhibits the highest activity because of its high phenolic content. Therefore, essential oils are suitable alternatives to chemical additives, satisfying the consumer demand for naturally preserved food products and providing good food safety.

#### 2.1.4. Composition Properties/Activities Relationship

Phytochemicals, natural extracts, EOs, and other natural additives are complex mixtures, with some chemical components being prevalent, and finally, these determine their activities and biological effects, as evidenced in Table 2. Some correlations have been established by the determination of the composition. For practical applications, gas chromatography/mass spectroscopy (GC-MSD) methods must be essential methods to test their quality and that of other natural additives as complex mixtures. By the contact and volatilization bioassays, minimum inhibitory concentration (MIC) and sporulation have been established. Testing of their biological actions against food-borne pathogens or on various kinds of spoilage fungi, etc., is also useful. A clear demonstration of the composition–activity relationship is absolutely needed to obtain reproducible results when a phytochemical, natural extract, or essential oil is scaled up for current practical applications. The composition properties/activities correlations have been established both for EOs and natural extracts. Their antimicrobial and antifungal activities have been tested against different types of spoilage fungi, such as *Fusarium graminearum*, *Penicillium corylophilum*, and *Aspergillus brasiliensis*, and potential pathogenic food bacteria, *Staphylococcus aureus*, *Escherichia coli*, and *Listeria monocytogenes*, using the disk diffusion method. The EO components of thyme, marjoram, and sage were analyzed by their phytochemical profile. It has been established that *C. nardus* and *C. citratus*, or *C. schoenanthus* combinations, exhibited a synergistic effect against *Aspergillus flavus* and *Aspergillus*, respectively. The combination of the antifungal agent, voriconazole, and the essential oil of *Pinus sylvestris* L. (*C. citratus* and *C. schoenanthus*) completely inhibited the production of the four aflatoxins [106]. This combination has the greatest antifungal activity against *Microsporum canis* in comparison with components tested alone [107]. The thyme EO has the highest inhibitory effect in comparison with clove and tea tree EOs, and they can be used as antimicrobial agents against fungi and bacteria.

The EOs used after nanoencapsulation, nanoemulsions, and nanocomposites showed an enhanced antibacterial and antioxidant performance [108].

The antioxidant properties of the samples were evaluated using total antioxidant parameters (such as total antioxidant capacity (TAC), ferric reducing power (FRP), antioxidant activity (AOA) towards 2,2-diphenyl-1-picrylhydrazyl (DPPH·), and total phenolics by the Folin–Ciocalteu method. The FRP was 46–321-fold lower than TAC, and they are dependent on the contents of phenolics identified in the samples. Terpenes, isopropylmethylphenols, and eugenol are the major components of all essential oils. The thyme essential oil had the highest antioxidant parameters, while sage showed the weakest antioxidant properties [109]. A study on the antifungal, antiaflatoxin, and antioxidant potential of the EOs extracted from *Mentha pulegium*, *Myrtus communis*, and *Mentha piperita* showed efficacy as a preservative, from which 387 fungal species have been isolated. The EOs inhibited the aflatoxin B1 production at lower concentrations [110]. The major components identified for the essential oil extracted from *M. alternifolia* are α-pinene (21.64%), γ-terpinene (21.09%), terpinen-4-ol (17.31%), limonene (9.37%), and o-cymene (6.54%) [111]. This essential oil showed the highest antifungal activity on *Aspergillus niger*, followed by *Aspergillus fumigatus*, *Aspergillus flavus*, and *Penicillium notatum*, respectively. The MIC values vary in large limits depending on EO composition and target microorganism (50 ppm for turmeric/*Bacillus coagulans* or 0.6 µL/mL lemongrass/*E. coli* to 45.0 mg/mL sweet marjoram/*E. coli)*. The ratio of MIC/minimum fungicidal concentration (MFC) values of *M. alternifolia* essential oil varied between 1.05 and 1.68 mg/L, showing that this oil has a lethal effect on all of the mold species.

The most common EOs that have been studied/tested in the last 5 years as active agents in polymeric materials include cinnamon (cinnamaldehyde) [112], rosemary [113], eugenol and ginger, oregano [114], citrus [115], thyme [116,117] *Zataria Multiflora* EO [118], eugenol and clove oil as stabilizers for rubber [119], and many others. Some correlations between composition and properties have been evidenced.

*Cinnamon oils* (*CEO*) *and extracts* [112], because of their major components eugenol and cinnamaldehyde, possess antimicrobial effects on both Gram-negative and Gram-positive bacteria like *Salmonella enterica*, *E. coli*, and *Listeria monocytogenes*. They act by degradation of the cell membrane of bacteria and also show significant inhibitory effects against fungi, such as *Laetiporus sulphurous*, *Coriolus versicolor*, *Eurotium* spp., *Penicillium*, and *Aspergillus* spp. Such EOs are useful in the food and cosmetic industries. The incorporation of medicinal herbs/plants in a polymer matrix has been investigated due to their antibacterial and wound-healing properties. The CEO decreased the moisture content, solubility in water, water vapor permeability, and elongation at break of chitosan films, suggesting a cross-linking effect of CEO components within the chitosan matrix.

*Garlic EOs* have strong antimicrobial activity towards foodborne pathogens, being an effective preservative for vegetables, meat products, fish, rice, fruits, and dairy products. A combination of chitosan (CS) and chondroitin sulfate into hydrogels through the freeze gelation process and enriched with garlic (Gar) by soaking the hydrogels in garlic juice led to faster wound healing and resistance to microbial growth at the wound surface and promoted tissue regeneration [120].

A study of the therapeutic effect of garlic extract evidenced diverse effects on mammalian cells, being either cytotoxic for them, especially for cancer cells, or protecting against oxidative stress. It induces the oxidative changes in the erythrocytes, thiol, and hemoglobin oxidation [121]. Polyethylene glycol (PEG)-coated nanoparticles loaded with garlic EO show insecticidal activity against *Tribolium castaneum.* Encapsulating garlic essential oil into solid lipid nanoparticles (SLNs) by the ultrasonic-solvent emulsification technique enhanced its insecticidal activity against potato tuber moth larvae, *Phthorimaea operculella* (Zeller) (*Lepidoptera: Gelechiidae*), effectively controlling these larvae.

EOs and extracts from *Coriandrum sativum* L. (*C. sativum*) provide antifungal, antibacterial, and antioxidative activities, having an indispensable role as a preservative and flavoring ingredient to prolong the shelf life of foods, and it is known by its therapeutic action as a pharmaceutical compound and is a component of the perfumes (lotions and fragrances) [122].

The essential components identified in the various *oregano essential oils* (OEO) are thymol and carvacrol, which produce a characteristic odor, antioxidant and antifungal antimicrobial activities, and lipid peroxidation in fatty foods, and also have antidiabetic characteristics.

*Thyme EO*, as a white crystalline substance with a pleasant aromatic odor and strong antiseptic properties, is useful as an aroma/flavor ingredient for culinary effects and also is an antioxidant, antimicrobial, antitussive, antitumoral, and spasmolytic ingredient for medicinal and cosmetic applications. Seven volatile compounds represent 98.838 ± 0.99% of the total [123,124,125]. The bioactive compounds from thyme EO are linalool, α-terpineol, camphor, caryophyllene, and γ-terpinene. The thyme EO has bacteriostatic and bactericidal effects, with both liquid and vapor phases being biodegradable and renewable, and environmentally friendly for use in indoor sanitation, the volatile compounds being very efficient. Thyme EO drastically reduces or even eliminates the presence of microorganisms typically found in bioaerosols and irreversibly affects the growth of the foodborne pathogens *S. enterica*, *Y. enterocolitica*, and *L. monocytogenes* [126]. Thyme EO improves bioaerosol levels and sanitizes surfaces. The MBC/MFC values of thyme EO 100% are significantly lower than positive controls (NaClO 3%, H_2_O_2_ 3%, gentamicin, vancomycin, amoxicillin, and tioconazole). The effect is mostly equivalent to or superior to chemical disinfectants [127]. Thyme EO is a promising candidate for use in nutraceuticals and pharmaceuticals. Thymol and carvacrol, due to their multifunctionalities and properties (antioxidant, anti-inflammatory, antiseptic, anticancer, antimicrobial, antibacterial, antifungal, antibiofilm, and antiviral) and antiparasitic (insecticidal, antimalarial, antiacaricidal (antimite and antitick), antitoxoplasmosis, and antileishmaniasis), are promising additives not only for the entire food chain but also for various applications [128]. Encapsulation helps to keep and improve their activities [129].

*Clove oil* (*Syzygium aromaticum*), with main components eugenol, eugenol acetate, caryophyllene, and α-humulene that contribute to its biological activity, is one of the most useful and beneficial spices used in agricultural, food, pharmaceutical, and cosmetic industries [130]. It exhibits extraordinary antibacterial activity against a huge number of microorganisms, being found very efficient. A combination of clove (40%), basil (20%), and thyme (40%) oil has a synergistic effect [131], reducing the doses required, costs, toxicity, etc.

An increasing interest for certain polysaccharides, metal–organic frameworks (MOFs), bioactive or active peptides (AMPs), enzymes, etc., with particular properties/activities to be used as BACs has recently become evident.

*Polysaccharides* found predominantly in plants (pectin, starch, and cellulose), aquatic animals (heparin, hyaluronic acid, chitosan, chitin, and chondroitin sulfate), and microorganisms (those from *Ganoderma lucidum*, xanthan gum, and alginate) exert antibacterial activity and have biological properties, such as immune-stimulating and anticancer properties, being useful in the food and medical industries as candidates for the creation and application of novel antibiotics. Microbial polysaccharides are essential to the development of vaccines for preventing infectious illnesses [132]; some of them have demonstrated superior immobilization performances [133,134].

Due to their antioxidant, antitumor, antidiabetes, antibacterial, immunomodulatory, neuroprotection, and organ protection activities, the *M. charantia bioactive polysaccharides* are the key functional ingredients. *Momordica charantia* L. of the family Cucurbitaceae, widely grown in Asia, Africa, and the Caribbean for its edible fruit, is a well-known medicine because of its high pharmaceutical and nutritional values; it has been used in various Asian and African herbal medicine systems [135,136,137].

*Metal–organic frameworks* (MOFs) as porous hybrid supramolecular materials are developing as active agents/additives to extend food shelf life and maintain food safety. Some examples of MOFs are cyclodextrin-based [138], single-walled nickel [139], electrospun pullulan/polyvinyl alcohol nanofibers incorporated with porphyrin [140], copper antiiterephthalate [141], etc. Chemically stable MOFs contain such metals in their structure: aluminum, iron, zirconium, titanium, copper, and zinc. In active packaging, the MOFs act as host–guest systems and active agents as oxygen scavengers, antimicrobials, moisture absorbers, and ethylene scavengers. MOFs serve as host molecules for controlled release of active organic molecules such as 1-methyl cyclopropene, hexanal, ethanol, etc. [142,143], also being potential candidates as gas absorbers and to create high-performance antibacterial materials. Pathogenic microorganisms pose significant challenges in food preservation, leading to postharvest losses, economic losses, and health risks. An effective strategy was developed for antibacterial food packaging using a multifunctional MOF nanocomposite composed of zeolitic imidazolate framework, tannic acid, and chitin nanofiber that inhibits pathogenic microorganisms’ growth and maintains fruit integrity, extending their shelf life [144].

*Bioactive or active peptides are* found in abundant natural primary origin sources (e.g., animals, plants, and marine) and in the edible insects (*Schistocerca gregaria*, *Tenebrio molitor*, and *Gryllodes sigillatus*). The research on insect-derived peptides is relatively new. They are extracted through various techniques, and they are mainly applied in the food industry to obtain functional and nutraceutical foods or can be used as additives to enhance solubility, emulsification, foaming, and water/oil binding capacity. Bioactive peptides show a variety of properties, such as antioxidant, anti-inflammatory, antidiabetic, antimicrobial, antihypertensive, and angiotensin-converting enzyme inhibitory activities [145]. In the composition of biodegradable packaging materials, such as the alginate-based films, the bioactive proteins positively affect the safety of food, prevent the development of oxidative and hydrolytic reactions during the storage of food products, and inhibit the growth of microorganisms and bacterial pathogens [146].

Bioactive peptides have no antimicrobial or antidiabetic properties. They are ideal ingredients in cosmetic formulations due to their potential to enhance skin health and beauty because of their various biological activities [147].

*Enzymes* are proteins that act as biological catalysts by accelerating biochemical reactions. The major sources of enzymes are microorganisms as bacteria, yeasts, and fungi, which produce a large number of enzymes used in food production, biofuel development, etc. Glucose oxidase is useful in different industrial processes in the production of biosensors, and in the food industry, it ensures the improvement of the properties of baking products, replacing chemical additives. Its stability is achieved by encapsulation and drying in certain conditions [148].

*Rennet* is an indispensable additive in cheese production [149]. *Saponins* play a vital role in the health benefits and biological activities [150]. *Asparagus* saponins are an effective, natural, beneficial ingredient in functional foods.

A short summary of the studies related to the composition–activity relationship for natural additives that is strongly dependent on the prevalent amount of certain components is presented in Table 2.

**Table 2:** Short summary on studies related to the composition–activity relationship for natural active additives.

Active Compounds	Activity and Application	Refs
Terpenes and terpenoids	Protect plants from predators or lure the pollinators. Carvone is used in anti-infective therapy; thymol, carvacrol, eugenol, and menthol are good alternatives to synthetic fungicides in the food industries due to their potent and wide antifungal activity, protecting agricultural crops from microbial infections. Therapeutic effects in neurodegenerative and cardiovascular diseases, cancer, diabetes, and aging processes. Terpenoid derivatives exhibit antimicrobial activity; composites demonstrated long-term antioxidant release activity. The antioxidative biocomposites are used in active protective packaging of both food (fruits) and cosmetic products.	[151,152,153,154,155,156,157,158,159]
Phenols and polyphenols (vanillin, gallic acid, eugenol, ferulic acid, carvacol, thymol)	Antibacterial activity, in vitro and in vivo animal tests demonstrated that they have antioxidant, anticancer, antibacterial, and antidiabetic capacity and health-promoting properties.	[152,153,154,160,161,162,163,164,165,166,167,168,169,170,171]
Flavonoids: flavonols, flavan-3-ols (catechins), flavones, and isoflavones, anthocyanins, with different heterocyclic structures	Strong antimicrobial action against a wide range of pathogenic microorganisms, Robust antioxidant and biological activities; useful in the pharmaceutical and health care industries.	[172,173]
Anthocyanins	Natural water-soluble colorants/pigments applied as safe food colorants; multifunctional properties such as excellent antioxidant, antimicrobial, and pH-sensitive properties; useful as pH colorimetric film indicators; potential health-promoting properties as antidiabetic and anticancer activities, and in cardiovascular and neuroprotective prevention.	[25,174,175,176,177,178,179,180]
Sulfur-containing compounds (allicin, ajoene, dialkenyl, dialkyl sulphides, S-allyl cysteine and S-allyl-mercapto cysteine, and isothiocyanates)	Antibacterial action against both Gram-positive and Gram-negative bacteria.	[181]
Coumarins	Chemical defense against predators, pathogens, and insects. Strong antibacterial action against *S. aureus*, *Salmonella typhi*, *Enterobacter cloacae*, and *Enterobacter aerogenes.* Potential therapeutic agents against several human diseases, anti-inflammatory, anticoagulant, antibacterial, antifungal, antiviral, antihypertensive, antituberculous, anticonvulsant, antiadipogenic, anticancer, and antihyperglycemic. Pharmacological activities, vasodilator, estrogenic, anticoagulant, analgesic, sedative and hypnotic, hypothermic, antihelminthic, antioxidant, and dermal photosensitizing activity, and neuroprotective actions.	[182,183]
Tannins	Water-soluble polyphenolic biomolecules. Applications as coagulants, adhesives, flotation agents, tanners, dyes, additives, or biomolecules	[184,185]
Lignins	Antimicrobial agents in packaging and textiles, antioxidants, adhesives, biosurfactants, reinforcing agents, compatibilizers, hydrogels, adhesives, anticorrosion agents, carbon fibers or carbon black, phenolic resins, flame retardants, foam composites, new biomedical materials, and cosmetics.	[186,187]
Alpha-tocopherol	Vitamin E subclasses. A type of lipid is a naturally occurring antioxidant.	[188,189,190]
Carotenoids (>600): β-carotene, lutein, astaxanthin, bixin, norbixin, capsanthin, lycopene, canthaxanthin, β-Apo-8-carotenal, zeaxanthin, β-apo-8-carotenal-ester, etc.)	Preclinical, clinical, and epidemiological studies showed their efficacy on cancer, obesity, type 2 diabetes, cardiovascular diseases, osteoporosis, neurodegenerative disease, mental health, eye, and skin health.	[191,192,193]
Alkaloids (piperine, berberine, quinoline, reserpine, sanguinarine, tomatidine, chanoclavine, squalamine)	A broad spectrum of antimicrobial activity and diverse bioactivities, such as bacteriostatic and bactericidal effects, are interesting for the food, pharmaceutical (drugs for therapeutic applications), and cosmetic industries.	[194,195]
Antimicrobial peptides	Active against a broad spectrum of microorganisms (bacteria, viruses, fungi, and protozoa).	[196,197]

*Terpenes and terpenoids*, *or isoprenoids*, are the largest class of compounds (more than 30,000 compounds) responsible for *the aromas*, *flavors*, *and colors* depending on the vegetation types. They are simple unsaturated hydrocarbons (C_5_H_8_)n (such as pinene, myrcene, limonene, terpinene, p-cymene, etc.), while terpenoids are modified terpenes with different functional groups as alcohols, aldehydes, or ketones, and oxidized methyl groups present in their chemical structure. Plants create terpenes to protect themselves from predators or to lure the pollinators. The most important are monoterpenes, sesquiterpenes, and monoterpene alcohols. Two isomers of β-citronellol are abundant in cannabis and many other plants, fruits, and herbs. They show inhibitory effects on *Candida* (*C. albicans* and *C. tropicalis*). β-citronellol acts on the cell membrane of the fungi, but not on the wall. Carvone is used in anti-infective therapy. Ketones and aldehydes are commonly found in plants, including ionone, zingerone, cinnamaldehyde, citral, and perillaldehyde. Cinnamaldehyde, as an *antibacterial agent*, has a broad spectrum. The encapsulation of these compounds into inorganic nanocarriers, such as nanoclays, prolongs the release and protects the compounds from harsh processing conditions [151]. Due to their *antioxidant behavior*, terpenes protect against different diseases, including neurodegenerative and cardiovascular diseases, cancer, diabetes, and aging processes [152,153,154,155]. Therapeutic potential for inflammatory diseases has also been demonstrated [156]. They interact with biological targets, being promising molecular scaffolds for chemical applications [157]. Eugenol and cinnamaldehyde, as terpenoids present in essential oils of several plants, are active against a wide spectrum of pathogens. Thymol exhibits synergistic activity toward all tested species of *Candida* in combination with fluconazole. Thymol and carvacrol, in addition to eugenol and menthol, are good alternatives to synthetic fungicides in food industries due to their potent and wide antifungal activity that can be used to protect agricultural crops from microbial infections [158]. Several terpenoid derivatives exhibited antimycobacterial activity, as the limonene-modified cellulose fiber-polylactic acid (PLA) composites demonstrated long-term antioxidant release activity. Eco-friendly additives are useful to develop sustainable biodegradable polyesters [159].

*Phenols and polyphenols* can be divided into different classes, depending on the chemical nature, such as phenols, phenolic acids (e.g., hydroxybenzoic acids and hydroxycinnamic acids), coumarins, flavonoids, quinones, tannins, lignans, lignins, and stilbenes [160,161,162,163,164], while from medicinal and edible sources many types of phenolic compounds have been obtained, such as flavonoids, bioflavonoids, phenylpropanoids, curcuminoids, coumarins, stilbenes, chalconoids, chromones, tannins, lignans, neolignans, phenolic acids and their glycosides, anthraquinones, quinones, xanthones, etc. *Polyphenols* are among the most significant bioactive by-products of the agri-food chain. Monophenolic, polyphenolic, and phenolic acid compounds from plants, foods, spices, insects, fungi, lichens, algae, and mammals inhibit or attenuate the initiation, progression, and spread of cancers in cells. Cinnamic and caffeic acids are simple phenols and phenolic acids with a single substituted phenolic ring, with one or more hydroxyl groups. Vanillin, gallic acid, eugenol, ferulic acid, carvacol, and thymol, found in plants, exhibit antibacterial activity attributed either to inhibition of critical microbial enzymes by reaction with sulfhydryl or other functional groups on the enzyme surfaces or to disruption of membrane integrity, altering the microbial metabolism, and inactivation of essential substrates required for microbial growth. In vitro and in vivo animal tests demonstrated their antioxidant, anticancer, antibacterial, and antidiabetic capacities, etc. Such health-promoting properties are studied for both research and commercial purposes, being active in the prevention of cardiovascular diseases. Dietary foods rich in phenolics proved to be protective against the development of osteoporosis and neurodegenerative diseases, etc. [165,166]. Some phenolic BACs, such as resveratrol, baicalein, biochanin A, formononetin, myricetin, quercetin, p-coumaric acid, curcumin, taxifolin, etc., play an important role in enhancing antibiotic activity against resistant pathogens.

Polyphenols as bioactive compounds exist in a wide variety of marine organisms, including algae, fish and crustaceans [167].

The antioxidant activity of polyphenols can be attributed to direct reaction with free radicals (primary antioxidants) or through reaction with chelating free metals (secondary antioxidants). Many phenolics can inhibit oxidative changes (such as lipid, phospholipid, or protein oxidation) due to their metal chelating and free radical scavenging properties [168]. Polyphenols and plant extracts rich in polyphenols have food applications to control oxidation processes in different food matrices [169]. Tea polyphenols are extensively used in antibacterial and antioxidant food packaging as in active films [170,171].

Polyphenols from fruits and vegetables are employed to create additives, additional foods, and supplements like natural preservatives and functional food ingredients due to their antioxidant and antimicrobial properties in the food, cosmetic, and pharmaceutical industries. In some cases, nanocarriers have been used to protect polyphenols during food processing, to solve the issues related to low water solubility, to transport them to the site of action, and to improve their bioavailability.

*Flavonoids* are abundantly found in edible and inedible plants, in both aglycosylated and glycosylated forms. Based on their level of oxidation and unsaturation, they have been classified into various groups as anthocyanins, flavonols, flavan-3-ols (catechins), flavones, and isoflavones, with different heterocyclic structures, etc. [172,173]. As secondary plant metabolites synthesized in response to microbial infection, they have a strong antimicrobial action against a wide range of pathogenic microorganisms and also have robust antioxidant and biological activities, being useful in the pharmaceutical and healthcare industries. Many flavonoids generate reactive oxygen species (ROS) (such as epicatechin, epigallocatechin gallate, and quercetin) that can damage important cellular processes.

*Anthocyanins*, as natural water-soluble colorants/pigments (may appear red, purple, blue, or black), which belong to the plant-based chemicals called flavonoids (e.g., 6000 flavonoids contain six main types of anthocyanins), have attractive features [25,174]. The color of flowers, fruits, and vegetative organs of many plants is explained by the presence of anthocyanins. Anthocyanins exhibit multifunctional properties and are applied as safe food colorants, excellent antioxidants, antimicrobials, and pH-sensitive properties as diverse colors at varying pHs, with biocompatibility being useful as pH colorimetric film indicators for stability under different storage conditions [175,176] and potential health-promoting properties as antidiabetic and anticancer activities and in cardiovascular and neuroprotective prevention [177]. Anthocyanins exhibit strong antibacterial activity against food-borne pathogens (e.g., *Salmonella enteritidis*, *Vibrio parahaemolyticus*, *Staphylococcus aureus*, *and Listeria monocytogenes*). Unfortunately, they can undergo oxidative degradation, thermal degradation, degradation caused by exposure to light, a change in pH, and enzyme activity. Stability of anthocyanins can be enhanced by the functionalization with acyl groups on the sugar moiety, the presence of metal ions, self-association, copigmentation, and encapsulation.

Phenolics, iridoids (a type of monoterpenoid in the general form of cyclopentanopyran), amino acids, alkaloids, and some polymers possess copigmentation ability. Copigmentation phenomena relate to the non-covalent interaction between anthocyanins and organic molecules (co-pigments) that produce hyperchromic and bathochromic shifts. It has been established that the non-covalent interactions protect the anthocyanin molecules from nucleophilic attack by water, while the copigmentation extends the π-conjugated systems, responsible for favoring π–π stacking interactions and hydrogen bond donor/acceptor groups such as –OH and C=O groups [178]. To avoid their inherent instability against certain environmental conditions, various cellulosic materials are used. The matrix/carriers, as anionic cellulose-derived polymers, can act as an electrostatic binding site for positively charged freshness biomarkers, being used as colorimetric food freshness indicators for meat, fruits, and milk [179,180].

*Sulfur-containing compounds* (e.g., S-allyl cysteine and S-allyl-mercapto cysteine, isothiocyanates, allicin, ajoene, dialkenyl, and dialkyl sulfides) exert antibacterial activities against both Gram-positive and Gram-negative bacteria [181]. Sulfur, as an essential plant macronutrient, has a pivotal role in plant disease resistance. Sulfur-containing amino acids (cysteine, methionine), glutathione, etc., have also been identified. The development of the concepts of sulfur-induced resistance (SIR) and sulfur-enhanced defense (SED) proved their roles.

*Coumarins* are chemical compounds in the benzopyrone class, found in many plants (>1300) such as cinnamon, vanilla, strawberries, cherries, apricots, green tea, peppermint, carrots, and celery, acting as a chemical defense against predators, pathogens, and insects and exhibiting strong antibacterial activity (e.g., against *S. aureus*, *Salmonella typhi*, *Enterobacter cloacae*, and *Enterobacter aerogenes*). They appear as a colorless or white crystal form or flakes. Coumarin has a pleasant and fragrant vanilla odor with a bitter, aromatic, burning taste and is the precursor for the warfarin drug because of its anticoagulant properties similar to vitamin K. The MIC varied from 5 to 125 µg/mL. Coumarin and its derivatives are potential therapeutic agents against several human diseases (such as anti-inflammatory, antihypertensive, antituberculosis, anticonvulsant, antiadipogenic, anticancer, and antihyperglycemic pharmacological activities, vasodilator, estrogenic, anticoagulant, analgesic, sedative and hypnotic, hypothermic, antihelminthic, antioxidant, and dermal photosensitizing activity, and neuroprotective actions). These are explained by selectively binding to proteins involved in various cellular processes with a beneficial impact on human health [182,183].

*Tannins* are water-soluble polyphenolic biomolecules, such as hydrolysable tannins, condensed tannins (the most dominant ones in the world market), and proanthocyanidins found in legumes, leaves, bark, fruits, and roots (herbaceous and woody plants), grapes, etc. [184]. Ellagitannins are bioactive polyphenols present in pomegranate. They form complexes with proteins, cellulose, and minerals. In hydrolysable tannins like gallotannin, hydroxyl groups are esterified with gallic acid or ellagic acid, whereas condensed tannins (proanthocyanidins) are composed of oligomers of the flavan-3-ols and other related flavanol residues. Major applications of tannins are in the leather, food, 3D printing, food supplement, biomedical, and health care industries, being used as coagulants, adhesives, flotation agents, tanners, dyes, additives, or biomolecules [185].

*Lignins:* Natural polyphenols, such as lignin, lignan, and tannins, are important structural materials to support tissues of plants, having a fundamental role in different stages of plant life. Lignins protect plants against attacking organisms. In industries, the lignins have a wide range of applications, such as adhesives, biosurfactants, antimicrobial agents in packaging and textiles, antioxidants, reinforcing agents, hydrogels, adhesives, anticorrosion agents, carbon fibers or carbon black, components of some phenolic resins, flame retardants, polyurethanes, foam composites, compatibilizers, and new biomedical materials, and they are also important in cosmetics [186,187].

*Vitamins:* Tocopherols (alpha-tocopherol-vitamin E), ascorbic acid (vitamin C), and carotenoids are important natural antioxidants in various fruits and vegetables. Encapsulated into biodegradable polymer matrices, they offer increased antioxidant and antibacterial activities for active food packaging films or to achieve the controlled drug release [188,189,190].

*Carotenoids* are pigments in plants, algae, and photosynthetic bacteria that produce different colors in plants, vegetables, fruits, archaea, and fungi. There are more than 600 different types of carotenoids. As BACs with antioxidant properties, they are of broad interest in food applications and human health [164,191]. They are either composed only of carbon and hydrogen as carotenes (e.g., lycopene and β-carotene) or oxidized carotenoids, xanthophylls (e.g., lutein), that have substituent groups with oxygen, such as hydroxyl, keto, and epoxy groups [192]. It has been demonstrated that the carotenoids (such as β-carotene, lutein, astaxanthin, bixin, norbixin, capsanthin, lycopene, canthaxanthin, β-apo-8-carotenal, zeaxanthin, and β-apo-8-carotenal-ester) are potential ingredients in various fields such as food, feed, nutraceuticals, and cosmeceuticals. Preclinical, clinical, and epidemiological studies showed their efficacy on cancer, obesity, type 2 diabetes, cardiovascular diseases, osteoporosis, neurodegenerative disease, mental health, eye and skin health.

*β-carotene-containing* bioactive coatings into biodegradable polymer matrices (as CS–gelatin/starch nanocrystals; soy protein isolate/PVA) have been elaborated [193] to obtain reinforced nanocomposites for food packaging. Practical use is limited due to high cost, low aqueous solubility, and the propensity to chemical degradation during food processing and storage. PLA films containing carotenoids (β-carotene, extracted from carrot), tomato (lycopene), and annatto seeds (bixin) showed better UV protection and oxygen barrier properties.

*Alkaloids* are heterocyclic nitrogenous compounds such as codeine and morphine. The most studied alkaloids are piperine, berberine, quinoline, reserpine, sanguinarine, tomatidine, chanoclavine, and squalamine, exhibiting a broad spectrum of antimicrobial activity by disrupting the microbial membranes. They have diverse bioactivities, such as bacteriostatic and bactericidal effects, which are interesting for the food, pharmaceutical (drugs for therapeutic applications), and cosmetic industries. The antibacterial properties of plant alkaloids are explained by their intercalation into the cell wall or DNA within microbial cells, inhibiting the adenosine triphosphate-dependent transport of compounds across the cell membrane, leading to impaired cell division and cell death. In Gram-negative bacteria, squalamine interacts with the negatively charged phosphate groups in the bacterial outer membrane, which leads to the disruption of the membrane [194,195].

*Lectins*, *peptides*, *and polypeptides*: Lectin structures have specificity for carbohydrates and the ability to bind other carbohydrates, being promising antiviral agents [196].

*Antimicrobial peptides* (AMPs) are low molecular weight peptides (<10 kDa) of 8–100 amino acids of cationic and amphiphilic nature (see above). They are produced by plants in response to external stresses and infections, being active against a broad spectrum of microorganisms (bacteria, viruses, fungi, and protozoa) [197]. Membrane disruption occurs by interaction of the lipid components in microbial cell surfaces, or cationic polypeptides, by electrostatic interactions with anionic microbial cell membranes.

*Scleroprotein*/*Keratin*: Animal by-products (defined by the European regulation EC No. 1069/2009) (e.g., non-edible tissues, feathers, wool, hooves, horns, claws, beaks, pig bristles, bovine hide hairs, and fish scales) are keratin-containing. Scleroprotein keratin, both in humans and animals, is the most abundant protein after collagen. Extraction of the keratins from animal by-products is achieved by chemical methods (such as acidic or alkaline hydrolysis, oxidation, reduction, and solvation in ionic liquids), biological methods (microbial and enzymatic), and new methods (such as ultrasounds, pulsed electric fields, high pressures, microwave-assisted extraction, deep eutectic solvents, steam flash explosions, etc.).

Keratin has a variety of functions, such as cohesion and structuring of tissues, waterproofing, temperature regulation, wound healing, nerve repair, cushioning to protect the deeper tissues against mechanical shocks and infection, and excretion of wastes and toxins from the integumentary system, etc. The applications of the keratin are as follows: agricultural products (e.g., biostimulants and coatings for fertilizers), functional foods (e.g., fiber analogs), bioplastics (anti-fire and antimicrobial coatings, edible casings), biomaterials (e.g., tissue and nerve repair and regeneration), pharmaceuticals (e.g., drug coatings for controlled release, anti-inflammatory products), cosmetics (e.g., hair and skin care), and textiles (e.g., functionalized wearables) [198].

*Fatty Acids:* Vegetable oils rich in unsaturated fatty acids are prone to oxidation, which produces free radicals [199,200].

Tocopherols, phenols, carotenoids, and chlorophyll derivatives exhibiting a bioactive effect have been identified in the composition of various oils. *Agave sisalana* can be considered to be a real alternative to the chemical plasticizers because it is biocompatible and biodegradable [201].

*Polymers*, *both synthetic and natural*, *especially biopolymers*, play important roles in advanced materials to enhance their properties and activities as both matrices and also as active additives/ingredients, offering a wide range of solutions in various applications. They can exhibit both already mentioned functions (antimicrobial, antioxidant, antifungal, therapeutic effects, etc.—Table 1, and also they can be designed/functionalized (by incorporating functional groups or other additives/ingredients) to show specific functions, such as optical, thermal, magnetic, conductive, or specific biomedical properties, etc. Their versatility allows them to be used in diverse fields, including agriculture, food packaging, medicine, environmental protection, energy storage, etc. Development of the advanced functional polymer materials is being driven by the fast-growing demand for new specific materials that can be used in innovative technologies to develop luminescent polymers, photovoltaic polymers, other electronic and optical polymers, biorelated polymers (particularly those for biomedical applications), supramolecular polymers, stimuli-responsive polymers, shape-memory polymers, separation polymer membranes, energy storage polymers, and covalent organic framework polymers. They are incorporated into a polymer matrix in order to improve processing, mechanical, thermal, electrical, and esthetic characteristics and can also provide antioxidants, anti-degradants, and antimicrobial activities, while some specialized compounds impart specific functionalities to the final product.

As it is well-known, the additives for polymeric materials can be divided into four categories: (1) functional additives (e.g., stabilizers that can ensure protection against weathering agents (UV radiation, oxygen and thermal stabilizers), antistatic agents, flame retardants, plasticizers, lubricants, etc.); (2) colorants for esthetic appearance of finished products (e.g., pigments, dyes): (3) fillers (e.g., CaCO_3_, talc, etc.): and (4) reinforcements (e.g., glass fiber, carbon fiber, plant-based fibers, etc.). The most commonly used additives in polymers are plasticizers, flame retardants, antioxidants, acid scavengers, UV lubricants, pigments, impact resistance, antistatic agents, and slip agents. Compatibilization agents for polymer blends also have importance in the processing and recycling of plastic materials.

Current trends in using polymers for food, water, and beverage applications consist of the design and preparation of the active packaging, bearing active ingredients in their formulation, and delivering chemical species such as antioxidants and antimicrobials that are released (or not) from the package to protect the goods inside and to extend their shelf life. Some polymers can serve as sensory polymers to detect contaminants as food quality indicators. In such cases, the interaction between the target species and polymer produces a measurable signal (change in color, shape, hydrophilicity, conductivity, etc.). An interesting trend is the use of edible polymers or biopolymers for food packaging for environmental reduction of waste. Edible films prepared from milk proteins (such as casein, lactoferrin, or whey protein) and polysaccharides are biodegradable, not toxic, and proven to be a promising alternative to polymers derived from oil and used in food packaging. *Active packaging* is based on natural polymers such as gelatin, starch, and chitosan, which are the most relevant environmentally friendly, biodegradable polymers obtained from natural resources, or synthetic polymers, such as poly(lactic acid) (PLA) or PP. In many systems, the modification of polysaccharides and other natural polymers, introducing different active additives in their formulations, is applied to improve their mechanical and thermal properties, decrease the permeability to water vapor, and increase antimicrobial properties, etc. Incorporation of the active agents onto material, surface modification, coating, immobilizing, etc., are efficient methods used to obtain high-performance materials with applications in various fields.

*Chitosan* is a biodegradable polysaccharide with antibacterial and antifungal properties, is marine-available, and is biocompatible. Natural additives rich in phenolic compounds are often included in the chitosan matrix in order to intensify the antioxidant properties of the obtained films. Chitosan nanoparticles (CS NPs), based on their different derivatives, were proposed as antioxidant and antimicrobial additives for active bioplastic packaging. Its antimicrobial activity, non-toxicity, and ability to form films make it suitable for enhancing food preservation and extending shelf life.

*Cellulose*, the most abundant natural polymer, is a good precursor for advanced functional materials due to its unique properties and versatility, especially as a nanomaterial. It’s being explored for applications in energy storage, flexible electronics, and biomedicine because of its renewability, mechanical strength, and tunable chemistry.

*Starch* acts as an “active” additive with antioxidant or antibacterial properties; when modified by oxidation, esterification, and etherification, it can prolong the food shelf life, serve as a carrier for bioactive compounds, or improve the functionality of edible films and coatings.

### 2.2. Nanoscale (Bio)Active Additives

*Nanoparticles* (NPs), *nanostructures*, *and nanomaterials*, because of their dimensions as small as 1–100 nm, are characterized by special physical and chemical properties, including particle size, shape, a high surface-to-volume ratio, high surface activity, particle-particle interaction, zeta potential, and molecular weight. Nanoparticle shapes are particulates, blades, platelets, spheres, cylinders, bricks, rods, wires, ribbons, tubes, scaffolds, beads, or sheets and fibers [202]. As it concerns the composition of the NPs as active additives, the following two types are known as inorganic and organic ones [203]:

(I) The *inorganic active additives are nanoparticles (NPs) made from inorganic substances as*: (i) *metallic*, such as silver, gold, or copper; (ii) *metal oxides*, such as ZnO, TiO_2_, SiO_2_, copper oxides (CuO, Cu_2_O), MgO, Al_2_O_3_, and iron oxides (e.g., Fe_3_O_4_, α-Fe_2_O_3_ hematite, γFe_2_O_3_ maghemite, FeMnO_3_); (iii) *nanocarbon-based*, including graphene, graphene oxide, carbon nanotubes (CNTs), fullerene, and quantum dots; (iv) *nanoclays* (mesoporous silica, nanoparticles of layered mineral silicates such as montmorillonite (MMT), cloisite, bentonite, kaolinite, hectorite, halloysite, laptonite, sepiolite, and organically modified nanoclays, etc. [204]).
(II) *The organic* NPs are: polymeric nanocapsules, micelles, liposomes, dendrimers, and nanobiopolymers such as cellulose nanowhiskers or nanofibers [205], starch nanocrystals, chitosan NPs and nanofibers, lignin NPs, DNA nanoparticles, and recently developed [206] two-component biopolymer NPs as sustainable lignin/chitosan. These are effective food coatings, promoting eco-friendly packaging, reducing waste quantity, and offering advanced bio-based solutions for food technology [207], etc.

The control of the nanoparticle properties is of great importance in many fields to test/keep reproducibility of the products and to improve their quality by reducing energy consumption and environmental pollution. As an example, such control is necessary for many pharmaceuticals (i.e., fat emulsions, liposomes, vaccines, hydrogels, etc.) to optimize the stability of dispersions and emulsions, thus improving the appearance, taste, and mouthfeel, and also to prolong the products’ shelf life, to guarantee the efficacy and safety of the formulations containing protein and polypeptide drugs (lysozyme, human serum albumin, immunoglobulin G, etc.), and to reduce and/or avoid immunological reactions and toxicity. Decreased size of NPs results in increased bioavailability or increased strength of a drug with the decreased dosage amount. Moreover, drug-loaded NPs show lesser toxicity and enhanced delivery, being suitable agents for the delivery of the functional nutrients to humans [208,209].

Plant-derived nanostructures and nanoparticles (based on proteins, polysaccharides, lipids, etc.) with diversified resources, and also inorganic NPs, are increasingly exploited for production and have applications in numerous fields. Nanoparticles from easily degraded materials, particularly liposomes, have been utilized for the COVID-19 vaccine in the delivery of sensitive mRNA. The delivery of the phytochemicals by nanocarriers is a candidate for targeted and bio-friendly therapy [210,211,212].

*Nanobiomaterials*, because of their impressive qualities, such as good biocompatibility, biodegradability, and high efficacy, are easily taken up by cells participating in normal cellular activities as biologically active molecules, assuring bio-renewability and sustainability of the useful nanostructures.

Nanostructures and nanoparticles promote the developments in various fields, such as (a) sustainable/precise agriculture, including improved crop yield, disease resistance, and nutrient uptake; (b) food and feed science, ensuring food preservation, packaging and the development of bioactive/smart packaging; (c) biomedical science, pharmaceutics, health care, nanostructured delivery systems for bioactive compounds, drug-gene delivery, cosmetics, textiles, food, adhesives, plastics, paper, (d) environmental applications, environmental remediation, environmental health, renewable energies [213,214], and so on. Moreover, their combination with inorganic nanoparticles opens interesting directions of study/application, because the synergistic effect leads to an improved technical performance of conventional bio-based materials, a.s.o. After coating cellulose nanocrystals (CNCs) with ZnO NPs, a significant increase in antimicrobial properties has been obtained. This could be a promising solution to obtain antimicrobial pharmaceuticals and natural food preservatives [215]. The other interesting results have been obtained by the combination of the thiol-bearing microcrystalline cellulose with Ag(NH_3_)_2_^+^ its use as a substrate for colorimetric detection of urinary cysteine, while AgNPs-impregnated bacterial cellulose with antibacterial *Moringa oleifera* leaf extract has been applied as an antibacterial dressing [216]. Cellulose nanocrystals and edible hydroxypropyl cellulose-based photonic materials encompassing the underlying self-assembly processes are developing green technology toward commercial application in a wide range of sectors [217].

## 3. Sources for (Bio)Active Compounds and Biopolymers

(Bio)active compounds (BACs) and biopolymers can be obtained from natural resources such as plants, animals, bacteria, fungi, microorganisms, and algae in aquatic mediums, including also seashell waste, seaweed, and eco-friendly and biocompatible by-products and biowaste from various processes [218].

By-products and biowaste of *animal origin* are buffalo horn, bovine Achilles tendon collagen, bovine hemoglobin hydrolysate, rainbow trout viscera, salmon fish, herring fish, poultry, etc. By-products and biowaste from *fruits* are grape peel and seeds, pomegranate seeds, pineapple peel powder, banana peels, banana inflorescence bracts, wine pomace, skin and seed extracts, orange by-products, wine pomace extracts and flour, pear stones, and vine–winery by-products; from *vegetables*, male flower *Musa paradisiaca*, soy milk, tiger nut milk, tomato by-products, overly ripe berries, and pomegranate peel; and from *cereals*, rice bran, *while those of dairy origin* are whey protein (such as whey casein)/water-soluble vitamins, lactose, etc.

Food biowastes resulting from global food processing industries are recognized as serious environmental and socio-economic issues when they are not properly managed, since these are prone to microbial spoilage. Usually, they are thrown into landfills or burned. They could release toxic pollutants and also create other ecological hazards, such as leachate production.

The existing global climate crisis, particularly in recent years, has led humanity to seek alternative ways to mitigate the disastrous environmental phenomena encountered and to search for solutions for biomass generated by human activities, considering it as an important resource for technological applications and the benefits resulting from biomass. The European Commission has elaborated an action plan to reduce food waste, integrating it into circular economy principles as a key strategy for the reduction, reuse, recovery, and recycling of materials and energy. Eco-friendly and biocompatible by-products and biowaste are rich in proteins, carbohydrates, lipids, and bioactive compounds (phenolics, dietary fibers, alkaloids, pigments, etc.), which must be recovered [219] and repurposed into value-added products for various industries, including food, biofuels, bioplastics, cosmetics, pharmaceuticals, textiles, active additives, etc. [220,221,222]. The starch and lignin are promising materials for sustainable and eco-friendly technologies [187,223].

Some agricultural practices contribute to soil degradation and air and water pollution. To avoid these, the agricultural waste is valorized by composting, production of animal feed, bioenergy generation, fiber extractions, biochar production, etc. Therefore, it is necessary to establish their interconnection and to establish some integrated solutions. Proper waste management through environmentally friendly strategies is required to reduce the environmental impact of date waste and to direct the transition of the date processing industry towards sustainability. The oilcakes, as by-products of the oil extraction process, are currently underused as animal feed, landfilling, or compost. The costs of drying and storage of the food processing residues are very high. In some cases, the fruit processing industries generate very diverse by-products and waste streams. Fruit processing by-products such as peels, seeds, and unused flesh are often utilized as fertilizers. Biopolymers derived from plant or animal sources are used in the development of bioactive films for fruits, vegetables, meats, and dairy products, offering antimicrobial and antioxidant benefits, being crucial in extending food shelf life, minimizing degradation, and protecting against oxidative and microbial agents [224]. Natural substances derived from agro-industrial by-products and waste (such as pomegranate peel, olive leaves, and grape vines) have antimicrobial and antioxidant activities, provide nutritional improvements without modifying organoleptic properties, and are promising active additives that could separately or synergistically extend the shelf life of fresh foods such as meat [225]. Pomegranate peel extract contains many bioactive compounds, such as tannins and flavonoids, showing interesting bioactivities, such as antidiabetic properties, regulating blood glucose levels, preventing cardiovascular diseases, reducing blood cholesterol levels, demonstrating anticarcinogenic activity, protecting lipids from oxidative damage, and having antioxidant and anti-aging properties, particularly beneficial for brain health as a natural alternative for the treatment of nociceptive and inflammatory pain [226].

### 3.1. Agro-Food By-Products and Aquiculture Waste

Many food by-products and sustainable agricultural wastes have gained considerable attention in recent decades because they are cheap due to their abundance, renewability, biocompatibility, and environmentally friendly nature; they also have remarkable biological features, etc. Processing residue/waste could be used for nutritious and healthy functional foods as a solution for the world food crisis. Approximately one-third of the world’s food production, i.e., 1.43 billion tons, is wasted annually, resulting in economic losses, contributing to greenhouse gas emissions, and negatively affecting food security and prices. The extraction of BC and biopolymers directly from origin resources results in a better-controlled composition and properties of the obtained products, but if huge quantities are used, a competition with the food industry will appear. To direct main activities to sustainability and environmental protection, the integration of by-products and biowastes is exploited. Secondary products derived from primary agro-food production/processing processes that could be recovered are a cheaper source of potentially biologically active ingredients (phenolic compounds, peptides, carotenoids, etc.), and they could be used as replacements for synthetic components. This approach contributes to food chain sustainability and system circularity. Valuable bioactive compounds, such as vitamins, proteins, polysaccharides, minerals, antioxidants, and aromatic oils, could be recovered from biowaste and used as functional ingredients, nutraceuticals, micronutrients, dietary fibers, lipids, pigments, minerals, or in biomedicine, pharmaceuticals, and cosmetic applications. Polysaccharides (xylans, fucoidans, claminarin, floridean, starch, and ulvans), vitamins, and minerals, as active compounds resulting from food processing by-products or agro-food by-products, have antioxidant, antimicrobial, antiviral, anti-inflammatory, immunostimulant, and prebiotic activities.

Oncom is produced from various raw materials such as tofu dreg, peanut press cake, and tapioca solid waste, applying various procedures involving various microbes, mainly *Neurospora* sp. or *Rhizopus* sp., and various processing steps. It is sensory-friendly and shows bioactivity capacities, including antioxidants, lowering cholesterol, cardiovascular disease prevention, etc. Therefore, Oncom might be an example of a potential sustainable food to overcome hunger and support the circular economy program.

From the processing of the vegetable agrifood (fruit peels and pomaces), many active ingredients result [220,227,228,229,230,231,232,233]., such as anthocyanins from grape skins, polyphenols, etc. The polyphenolic extracts of *Vitis vinifera* L. could be useful in the treatment of biofilm-related infections, often multi-resistant to antibiotics, if used as therapeutic adjuvants.

The by-products are rich in biopolymers such as polysaccharides (starch, cellulose, and pectin), proteins, and bioactive compounds. The food industry could use the vegetable waste powders as coloring and flavoring ingredients or natural preservatives, or they can be used to reformulate processed foods in order to improve their nutritional properties. The main wastes, by-products, and algae are recovered to achieve sustainable systems through the extraction of valuable compounds or waste treatments [234,235,236,237].

Demand for fresh and processed fruits has increased because of their health benefits and the increased world population/consumers. Processing of vegetables and fruits generates a substantial amount of residues as agri-food wastes in the form of peels, grain, seeds, pulps, pomaces, and stones/pits, posing a serious environmental and economic problem. Such wastes can be used as secondary starting materials to produce value-added goods, either to produce building blocks for bioplastics manufacturing or biofillers [238] within the principles of the circular economy.

For example, the quantity of the biowastes could reach 20–40% of processed materials. Therefore, the annual grape and wine processing generates around 5–9 million metric tons of solid waste, and canning and freezing of fruits and vegetables generate approximately 6 million metric tons of residues [239]. Grape production generates substantial agricultural waste, particularly grape pomace, a by-product rich in bioactive compounds. Fruit and vegetable biowaste reuse is strongly recommended to prevent environmental issues. Grape pomace is rich in fiber, lipids, sugars, proteins, and phenolic compounds, which makes it a promising substrate for Pleurotus mushroom cultivation [240]. This innovation supports the broader goals of sustainable agriculture because of the recycling of agricultural by-products, minimizing waste, and adding economic value. The BACs from these are rich in valuable compounds; the peels of citrus fruits, grapes, and apples, and the seeds of mangoes, avocados, longans, and jackfruits have been reported to contain >15% more phenolics than in the pulp [241]. The composition of grape pomace consists of 30% neutral polysaccharides, 20% pectic acid derivatives, and 15% phenols. The by-products of winemaking found applications as: food additives, food supplements, animal feed, nutraceuticals, and sanitizers, antibiotic adjuvants, thanks to their high polyphenol content; mainly resveratrol, tannins, anthocyanins, phenolic acids, and flavanols are the main BACs, which also are responsible for antibacterial activity [242]. By processing of vegetable and fruits as pulps, peels, grape skins, seeds, pomaces, bagasses, and stones/pits/cellulose can be obtained essential oils, starch, pectin, proteins/polyphenols, (anthocyanins, pigments), dietary fibers; cereals brans, husks, brewers’ spent grain, and corn cobs/starch, cellulose, hemicellulose, lignin/phytosterols, minerals, vitamins, etc. Packaging prepared using by-products from fruit and vegetable processing wastes offers a feasible alternative to reduce the production cost of edible films and coatings and to add value to food by-products [243]. Date fruit pomace is an abundant by-product of the date syrup industry, currently underutilized, being either fed to animals or landfilled. In a landfill, it ferments and raises serious environmental issues. This waste bioresource, rich in dietary fiber and phenolic antioxidants and having a high nutritional value, can be used as a dietary supplement, while its sugar content can be used to produce value-added biochemicals via fermentation. Banana peel is a sustainable feedstock for the extraction or synthesis of biologically active compounds, biopolymers, and nanomaterials useful in the agri-sector and aquaculture in pest/disease prevention, fertilizers, agrochemicals, bio-stimulants, and nutrient-delivery applications [244,245]. Hexavalent chromium Cr(VI) ions have been removed from water using pomegranate peel powder that has an excellent removal effectiveness of 50.32 mg/g [246]. Great efficacy in the treatment of chemical industrial effluents by using pomegranate peel powder as an abundant, cost-effective biowaste source is important because the UN SDGs (UN Sustainable Development Goals) have been achieved as the primary objective.

Ultrastrong fibers have been isolated from the vascular tissues of the *Senna alata* (L.) *Roxb*. (Candle bush) plant stem. These have mechanical properties comparable to those of commercially available natural fibers, being suitable for applications in door panels, furniture, and structural components. These fibers have excellent heat stability, and they are useful in insulation, circuit boards, dielectric substrates, and switches. And also, due to their antimicrobial properties, they have been found suitable for use in the packaging, textile, pharmaceutical, and wound dressing sectors [247].

Humic substances (HSs) are produced through a specific solvothermal synthesis directed to extract HS from the commercially available compost produced by a Sardinian firm, Verde Vita (s.r.l.), through the oxidative degradation of biomass derived from the organic fraction of urban or agro-industrial waste. They exhibit larger chemical stability under aerated conditions and lower cost. Humic substances (HSs), derived from biowaste oxidative processes, resulting in a refined product, exhibit intrinsic antioxidant and antimicrobial features [248] with potential applications like soil remediation, pollution treatment, and agriculture.

### 3.2. Residual Materials from Cereals

After processing the cereals by the milling and brewing industries, large amounts of by-products result, such as brans, husks, brewers’ spent grains, and corn cobs, which are highly nutritious. Cereal by-products include a lot of BAC as lipids, proteins, minerals, vitamins, phytosterols, polyphenols, and also starch and dietary fibers as hemicellulose (β-glucans and arabinoxylans), cellulose, and lignin. The rice bran and cereal dust are valuable yet underutilized by-products of grain processing. They can be bio-converted into bacterial cellulose by inexpensive solid-state fungal fermentation as a circular economy solution to reduce waste and convert it into by-products to enhance the sustainability of the cereal industry [249].

Palm oil production yields a valuable palm fatty acid distillate as a rich source of vitamin E and a multifunctional ingredient in the food agro-industry. Valorization of this distillate in the food sector assures sustainability [250].

### 3.3. Residual Materials from the Meat Industry

Residual materials from the meat industry are organs such as skin, blood, bones, meat, trimmings, fatty tissues, horns, hoofs, feet, skulls, feathers, tripe, liver, lungs, hearts, kidneys, and tongues, which contain collagens and also gelatin/vitamins, polyunsaturated fatty acids, etc. Gelatins, derived from collagens, which are abundant in animal skin, bones, and hooves, are used as gelling, stabilizing, thickening, and texturizing agents in confectionery, yogurt products, and dessert creams. Most commercial gelatins have mammalian origins, mainly pigs and cattle; other sources include fish and even chicken feet [251]. Gelatin is an important biopolymer used for edible packaging. Because of its hygroscopic nature, the combinations with other biopolymers are necessary to improve the functional properties of packaging and the shelf life of food products.

### 3.4. Residual Materials from the Dairy Industry

Dairy by-products contain large quantities of proteinaceous waste, specifically caseins, water-soluble vitamins, and lactose. The cheese sector produces the highest quantity. The whey is recovered as valuable coproducts (~50%), such as whey powder, protein concentrates, isolates, and lactose with multiple applications (foods, nutritionals, and pharmaceuticals). Caseins are the most abundant group of proteins, as bovine milk contains ~80% of the total protein [252]. Milk proteins are ideal for the production of biomaterials since they have good barrier and filmogenic properties. However, a large volume of residual materials from the dairy industry is discarded daily without any prior treatment.

### 3.5. Residual Materials from Algae and Marine Biowaste

The biopolymer production is based on the raw materials, generally from land-based crops, competing with the food sector. Aquatic biomass sources such as marine plants or seaweeds are alternatives for bioplastic production due to their fast growth rate, CO_2_ fixation, high density of biopolymers contained, and ability to withstand harsh environments. The seaweed industry for human consumption is growing. Biowaste from discarded fish scales and seashells is a rich source of natural polymers and other materials, such as collagen, keratin, and calcium carbonate, useful to produce eco-friendly, bioactive, biodegradable, and edible polymer materials and composites [253,254].

The algae used to obtain the bioplastics for packaging contribute to minimizing resource consumption, waste generation, and regulating greenhouse gas emissions. Macroalgae are a source of proteins (5–47%), lipids (1–5%), polysaccharides (15–76%), and minerals (7–36%). As a source, biopolymer/BC can be considered marine (fish head, trimmings (skin, fins, bones, scales, and muscle), viscera (liver, kidney, and roe)/myofibrillar proteins (collagen, gelatin)/omega-3, peptides, protein hydrolysates, iodine, vitamin D, Se, P, Ca; (crustacean shells/chitin/astaxanthin, CaCO_3_; algae biomass after extraction or others/carrageenans, agar, alginate/omega-3 mainly EPA (eicosapentaenoic acid) and DHA (docosahexaenoic acid), polyphenols, and pigments (β-carotene, astaxanthin, and fucoxanthin) [255,256]. Polysaccharides from seaweeds are employed in various industries as hydrocolloids; the most prevalent are alginates (brown macroalgae), carrageenans and agar (red macroalgae), fucoidans and laminarin (brown macroalgae), xylans and floridean starch (red macroalgae), and ulvans and (green macroalgae) agarose, which involves numerous different phytonutrients in the outermost layer of the cell and is rich in sulfated polysaccharides (SP). SP-based nanomaterial has an enhanced potential value in the nanotechnology field. Many interesting compounds, including pigments and polyphenols possessing biological and biochemical functions, have been extracted from seaweeds. Seaweed, or marine macroalgae, is an underexploited natural source of polysaccharides, although it has extensive applications in different industries, nutrition, food quality, and retaining characteristics of supplements and biomedical applications. *Ulva lactuca*, known as sea lettuce, is a green macroalgae powder that acts as an active additive in crab (*Portunus segnis*). Chitosan-based multifunctional (antioxidant, antimicrobial, renewable, and biodegradable) films for natural active food packaging, biomaterials, biostimulants, biorefineries, etc., offer significant economic, environmental, and social benefits. The by-products are applied as filler and fiber reinforcements, food and feed supplements, fertilizers, cosmetics, pharmaceuticals, and epoxy resins [257]. The sulfate polysaccharide NPs extraction from macroalgae is extensively applied in various fields of daily life. Four different species of agar-producing red seaweeds (*Gelidium corneum*, *Gracilaria chilensis*, *Gracilaria tenuistipitata*, and *Gracilariopsis longissima*), minimally processed by melt blending combined with compression molding, led to films with good quality [258]. Cyanobacteria and microalgae have been tested for bioplastic production as polyhydroxyalkanoates (PHA), which are naturally accumulated by microalgae. PHA-based materials are biodegradable, but they are not edible [259].

Marine-originated biomaterials such as seaweeds, marine sponges, arthropods, cnidaria, mollusks, and also alginate, vitamins, laminarin, collagen, chitin, chitosan, gelatin, hydroxyapatite, biosilica, etc., have very interesting biomedical applications due to antimicrobial, anti-inflammatory, anti-aging, and anticancer effects [260]. Products based on chitin and its derivatives are used in chemical, agricultural, medical, cosmetic, textile, paper, and other industries. The veterinary, biomedical, beverage, sanitary, health, and personal sectors, as well as the catalysis, chromatography, sewage treatment, and biotechnological industries, all show great interest in marine chitosan and collagen, the most attractive bio-resources, because of their distinct biological properties [261].

### 3.6. Other Resources

Herbal medicine uses many of the fungi, including *polypores* isolated from *Aphyllophorales* such as *Ganoderma lucidum*, *Laetiporus sulphureus*, *Trametes versicolor*, *Grifola umbellata*, *Inonotus obliquus*, *Wolfiporia cocos*, etc., to isolate biologically active natural products. This large group of terrestrial fungi of the phylum *Basidiomycota* (basidiomycetes), together with *Ascomycota*, is the major source of pharmacologically active substances that display antiviral, cytotoxic, and/or antineoplastic activities. Numerous compounds isolated from polypores, often called biological response modifiers, showed phytotoxic, immunomodulatory, analgesic, cardiovascular, antidiabetic, antioxidant, insecticidal, and other activities. They are high molecular weight compounds, or immunopotentiators, that prevent carcinogenesis and tumor metastasis and show direct anticancer effects. About 75% of polypore fungi show strong antimicrobial activity, being considered a good source for developing new antibiotics. Polysaccharides derived from the fungal cell walls are interesting for their immunomodulatory activities, resulting in antitumor effects. Some proteins bound to polysaccharides from polypores and other basidiomycetes have found their market in Japan as anticancer drugs [261,262].

## 4. Developments in Extraction and Processing Methods to Assure the Quality/Activities of the Active Additives

### 4.1. Extraction

The selected technique for active additive extraction is critical for their final use. To obtain bioactive compounds from plants (as leaves, roots, stems, fruits, seeds and peels or other sources), the following conventional methods can be applied: maceration, percolation, decoction, hydrodistillation, steam distillation, hydrodiffusion, solvent extraction, Soxhlet extraction, flowering, maceration, and cold pressing.

**Figure 2:**
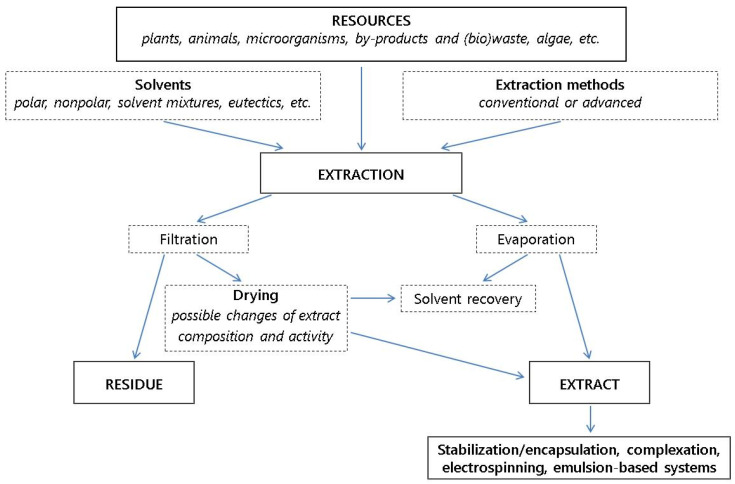
Flow diagram of the steps involved in the extraction and encapsulation applied for natural AC from different resources.

The steps involved in natural AC extraction and stabilization are schematically presented in Figure 2. They imply selection of solvent and extraction method and well-known operations of filtration, evaporation, drying, solvent recovery, and residue recovery.

Unfortunately, during the extraction process occurring at prolonged extraction times, high temperatures, and pH, chemical alteration of the composition and quality of EOs is possible because of hydrolysis, isomerization, and oxidation. Advanced methods for the extraction of active additives/EOs, such as supercritical fluid (CO_2_, propane, and argon), high hydrostatic pressure, pressurized liquid extraction, solvent-free microwave-assisted extraction, subcritical fluids (water) extraction, ultrasound-assisted extraction, high-voltage electrical discharges, power-pulsed electric fields, enzyme-assisted, and membrane-assisted prepurification, are considered the most promising techniques, being cost-effective and environmentally friendly due to short extraction time, low energy consumption, low and green solvent use, and less CO_2_ emission [263,264]. New green and environmentally friendly technologies are developing, such as supercritical/subcritical fluid extraction, negative pressure cavitation-assisted extraction, hot water extraction, high-pressure assisted extraction, etc. [264,265,266]. Each method has both advantages and limitations—Table 3.

**Table 3:** Advantages and limitations of the conventional and modern extraction methods applied for natural active additives, adapted from Refs. [264,265,266].

Method	Advantages	Limitations
**Conventional methods**	Conventional methods are still used to extract fragrance and aroma oils from plants due to their simplicity	Disadvantages associated with conventional solvent extraction techniques include prolonged extraction time, substantial amounts of solvents, and many extraction steps Thermolabile phytochemicals decompose or degrade during heating
Maceration	Simple extraction method, filtration or decantation applied to thermolabile AC	- Minimize solvent loss through evaporation; low efficiency and long duration of extraction
Digestion	Slight warming in the extraction process (35 to 40 °C)	- Avoid the temperature altering the bioactive; - Increased efficiency
Infusion and percolation	Easily soluble constituents; phenolic compounds were extracted from fruits, phenols, and flavonoids in boiling water for 10 min-infusion; a water–alcohol solvent mixture; aqueous HCl; extra efficient extraction	- The extract is termed leachate
Soxhlet extraction	- Continuous extraction procedure; - Ethanol or methanol as solvents - Enhanced yield is obtained compared to maceration	- The higher temperature risks the degradation of thermolabile compounds. - The amounts of both polyphenol and alkaloid extracts decreased with respect to maceration methods
**Modern techniques**		
- Accelerated solvent extraction (ASE)	High solvent temperature and pressure mark the ASE operations’ favorable conditions; better recovery of lipophilic and hydrophilic phytochemicals	- Temperature variation significantly impacts the extraction efficiency of different bioactive compounds
Supercritical fluid extraction (SFE)	- An increased temperature and pressure for a preset time (80 °C); the pressure of 20 MPa and the extraction time of 10 min favor extraction due to an increased diffusion coefficient and lowered viscosity. - Plant major compounds extracted: EOs, phenolics, alkaloids, phenolic acids and flavonoids, carbohydrates, carotenoids, pesticides, lignans, lipids	The highest phenolic acid content yield was achieved with high temperature, whereas lower temperatures afforded more efficiency in extracting high yields of flavonoids In some cases, Soxhlet extraction is preferred
Microwave-assisted extraction (MAE): incorporate microwaves and solvents during the extraction process	- A good preservation of the biological activities of the extracts; Green tea extraction: improved antioxidant activity of the phytocompounds and improved total phenolic content and color quality of the extracts - MAE is a rapid procedure, uses a low quantity of solvent, is economic, and has a higher extraction yield.	The viscosity of the solvent affects the extraction process, and also the solvent and plant material dielectric susceptibility affect the energy used in MAE
Ultrasound-assisted extraction (UAE)	Ultrasound-assisted extraction is an environmentally friendly, green, and cheap technique compared to conventional techniques, recovering green extracts in concentrated form without residual solvents, impurities, or defects - Higher yields of phenolics at a lower frequency of 40 kHz than at 120 kHz - Lowered the energy consumption and increased the recovery of phytochemicals, preserving their activity - The UAE method enhances the extraction speed and requires a relatively lower quantity of the solvent. - Using ionic liquids and deep eutectic solvents instead of conventional organic solvents, assisted by ultrasound extraction, improves the process	- Ultrasounds modify and disrupt physical and chemical characteristics of the material to be extracted
Supercritical fluid extraction: carbon dioxide	- The technique involves the solubilization of extractable chemicals and their separation - CO_2_ has low toxicity, readily available, and cheap - High yields and preserves the biological activities of extracts	- No trace of moisture is very significant, as moisture may adversely affect the yields - The equipment is expensive and difficult to clean, rendering the production uneconomical
Enzyme-assisted extraction: - aqueous extraction (EAAE) and cold pressing (EACP).	- Specific enzymes used: cellulase, pectinase, amylase, etc. - Non-extractable phytochemicals (pectin) accessible to the solvent - Is utilized in conjunction with other extraction techniques - Some enzymes are vulnerable to extraction	- The selected enzymes’ catalytic properties, mode of action, and optimum operational conditions should synergize
Pressurized hot water extraction	- An environmentally favorable technique affording considerable yields - Short extraction time, better quality extracts, and a cheap solvent - Elevated temperatures increased the recovery yield of phytochemicals - Very green at preserving the bioactivity of extracts	- Prolonged extraction times and high temperatures deteriorate the extracted phytochemicals

Recent studies are directed to achieve good economic viability, environmental friendliness, shorter extraction times, and better yields of ACs without compromising their biological activities. The advantages offered by modern techniques are higher yields for less solvent and energy demand, reduced required time, and better preservation of biological activities. However, it is still difficult to conclude about the choice of extraction technique, targeted phytochemicals, economic viability, environmental impacts, etc.

EOs are mainly extracted by methanol, ethanol, acetone, acetic acid, water, and others. Ethanol and water are the most adequate solvents, as they have GRAS status. The quality of EOs and natural extracts, their biological and physico-chemical properties, and antioxidant/antibacterial activities depend not only on the quality of the source (e.g., geographic origin, nutritional aspects, and storage) but also on the applied technology for their extraction. For example, the supercritical carbon dioxide extraction method of the *Rose geranium*, *Eugenia caryophyllata*, clove buds, and *Marchantia convolute*, has been compared with the hydrodistillation method, and an aromatic oil with superior performance and pharmacological activities has been obtained, while the carrot essential oil obtained by the supercritical fluid extraction method showed better antibacterial and antifungal properties against *Bacillueles cereus* as compared to the oil obtained by hydrodistillation. Modern techniques are very fast, very efficient, replicable, green, and more convenient, and they do not require a lot of energy.

*Alkaline extraction:* Polysaccharides isolated by alkaline extraction (0.5–4 M KOH) are enriched in pectinic polysaccharides (80.6–86%), namely rhamnogalacturonan and arabinogalactan, and hemicellulosic polysaccharides (13.9–19.4%). The short-chain fatty acid profiles indicated a high amount of lactic acid, followed by acetic and propionic acid [267].

Pine wood residues are transformed into bio-oil and separated into value-added chemicals. In this case, an integrated and innovative methodology was applied, including adsorption chromatography, vacuum distillation, liquid–liquid extraction, and precipitation at an industrial scale, allowing the simultaneous recovery of multiple chemicals [268]. The following three fractions with high recovery yields have been obtained: (1) an acid-rich fraction (containing acetic acid, formic acid, acetol, and furfural); (2) a sugar-rich fraction (e.g., levoglucosan, 5-hydroxymethylfurfural, and methyl cyclopentenolone); and (3) an antioxidant fraction, containing phenolic species with antioxidant properties (such as phenol, guaiacol, catechol and vanillin). Therefore, it has been demonstrated that it is possible to develop biorefineries and the integrated production of chemicals from a renewable feedstock such as wood.

### 4.2. Processing Methods and Encapsulation

Traditional methods of food preservation, including refrigeration, pasteurization, and using low pH, are not very efficient in controlling food pathogens. To inhibit spoilage and pathogenic microorganisms, the foods are heated at temperatures of 60–100 °C for seconds to minutes. These processes are efficient, but they can cause undesirable changes in food quality, loss of vital nutrients, high energy costs, and the loss of valuable resources. To avoid these, some techniques with minimal processing are preferred, such as ozone treatment, vacuum drying, osmotic dehydration, dense phase carbon dioxide treatment, and high-pressure assisted freezing. Non-thermal preservation methods consist of physical methods such as ultrasonication, pulsed electric field, pulsed light, cold plasma, high-pressure processing, ultraviolet, microwave, electrospinning, and utilizing natural preservatives isolated from animal, plant, or microbial sources, and these solutions are preferred because of consumers’ demand for a more sustainable and healthy food supply.

The associated drawbacks with the use of many BACs are their instability against environmental factors, heat, oxygen and light during storage, or unacceptable flavor profiles; poor dispersibility in food matrices; and food matrix effects, such as phase partitioning or interactions between ingredients and with food components (e.g., carbohydrates, lipids and proteins), thereby reducing their interactions with microbes and affecting their bioavailability and bioactivity.

Low solubility, bioavailability, and loss of activity can be minimized by *micro-*/*nanoencapsulation* for a safer application. Micro-/nanoemulsions are applied to flavors as limonene, EOs, and resins and to bioactive additives as antioxidants, preservatives, vitamins, minerals, colorants, enzymes, probiotics, lactic bacteria, etc. A combination of natural preservatives with different food/cosmetic preservation systems, emulsions, coatings, or micro- and nanoencapsulation has been tested to assure safety and non-toxicity of the materials [269,270,271]. Wall materials for nanoencapsulation of BC are natural (albumin, gelatin, casein, chitosan) or synthetic polymers such as poly (Ɛ-caprolactone), poly (lactic-co-glycolic-acid), polyethylene glycol (PEG), polylactides (PLA), polyglycolide (PGA), polyanhydrides, polycaprolactone, polyglutamic acid, polymalic acid, poly(*N*-vinylpyrrolidone), poly(methyl methacrylate), poly(vinyl alcohol), etc. The incorporation of nanoemulsified EOs into biodegradable materials has the double advantage of minimizing the concentration of active agent required to perform a valuable antimicrobial activity and reducing their sensory impact. All of these advantages are enhanced by reducing the size of (nano)particles that determine increasing the area per unit volume, and in this way the antimicrobial nanoemulsions of various types are developing as (a) the oil phase itself is antimicrobial (like an essential oil); (b) lipophilic antimicrobial (like a polyphenol) is dissolved in an inert oil phase (like corn oil); (c) an antimicrobial emulsifier is used to form and stabilize the nanoemulsions; and (d) the nanoemulsion is a combination of different antimicrobials to obtain a strong potency.

The main obstacles for using EOs as food preservatives, for pharmaceutical purposes, and in cosmetics are their safety limits, marked organoleptic effects, and possible contamination by chemical products, such as pesticides, toxicity, volatility, and hydrophobicity, that limit their use in their pure form, so their exploration and development are still a challenge [271]. It is also a possible diversity of composition varying with region, conditions of growing [272], etc.

As a result, encapsulation technologies, emulsion-based systems, and oil-in-water or water-in-oil micro-, nano-, and double emulsions are being developed and employed [208]. Types of morphologies obtained by encapsulation are mononuclear, multinuclear, multilayer, matrix, and irregular.

#### 4.2.1. Emulsion-Based Systems

The simple emulsion is the most common carriers for bioactive components. Emulsion-based systems are particularly interesting as colloidal delivery systems because they can easily be created from active ingredients using relatively simple processing protocols. In recent studies, bio-based novel multiple emulsions (MEs) and Pickering emulsions (PEs) have been studied as innovative colloidal delivery systems for controlled release of bioactive compounds and stabilization of the biopolymer films, such as those of starch with bacterial nanocellulose [273,274,275], which could be fabricated by relatively scalable and simple operations. Both are stabilized by food-grade particles, have health-promoting aspects, and are able to host the “clean label” and “green label” attributes. Emulsion-based encapsulation uses an emulsion as the core technology to trap and protect active ingredients, allowing for their stability, controlled release, and protection from degradation. They involve trapping the bioactive agents within small particles with a core (BAC)-shell (wall) structure designed to increase the water dispersibility, resistance to environmental conditions (light, oxygen, water), minimize interactions with nonspecific targets, maintain potency of antimicrobials, mask undesirable flavors, control the release profiles of the antimicrobials, and enhance handling and flow properties. The core of capsules contains some ingredients to be protected (BAC), and the wall material, also denominated as the coating, shell, membrane, carrier, or coat, is the external layer or layers that cover the core, made of food-grade materials (e.g., polysaccharides, proteins, and lipids). The following morphologies are obtained: mononuclear, multinuclear, multilayer, matrix, or irregular [276,277]. Encapsulation decreases volatility and increases chemical and thermal stabilities; protects against oxygen, light, pH, moisture, and gastric digestion; masks unpleasant taste and aroma; promotes controlled release; improves the solubility of lipophilic compounds in aqueous media; enables the prolonged absorption of nutrients; and may confer and facilitate the development of packaging materials with antimicrobial and antioxidant potential. MEs (e.g., double emulsions) have a variety of functions in many areas, such as food, cosmetic, and pharmaceutical industries, and separation sciences. The most common carriers for encapsulation of bioactives are still simple emulsions. MEs and PEs as advanced systems have been introduced as innovative colloidal delivery systems for encapsulation and controlled release to overcome limitations and improve stability, bioavailability, etc. Double emulsions obtained with low molecular weight (LMW) surfactants showed various types of instability mechanisms, e.g., coalescence, flocculation, and creaming. The delivery systems increase the solubility of phytochemicals, nutraceuticals, and food additives [278]. Polyphenols, due to their oxidizing ability, protect the materials from the damaging effects of ROS, acting as active additives preventing oxidative stress. The loading of polyphenols into lipid nanocarriers (NLCs) led to increasing bioavailability, reducing degradation, and protecting the antioxidant activity of the polyphenols. The NLCs are biodegradable and have no significant toxicity. The nanoemulsion, liposome, phytosome, solid lipid nanoparticles (SLNs), NLCs, and lipid-polymer hybrid nanoparticles (LPHNs) can encapsulate polyphenols to improve their biophysiological target [279], enhance polyphenol delivery, and promote their absorption and bioavailability.

#### 4.2.2. Nanoemulsions

Nanoemulsions are optically clear colloidal dispersions of two immiscible fluids, one of them being dispersed as small particles (d < 200–100 nm) in the other one. Nanoemulsified plant-based preservatives improve the food quality and safety in the meat, fish, dairy, and fresh produce areas [28,280,281,282].

Oil-in-water nanoemulsions are thermodynamically unstable systems but kinetically stable colloidal dispersions containing lipid nanoparticles dispersed in water, and they are manufactured from food-grade ingredients using mixing and homogenization. By varying oil droplet properties, such as composition, concentration, interfacial properties, and physical state, the physico-chemical properties, stability, and efficacy of antimicrobial nanoemulsions can be altered. Their breakdown occurs by different destabilization mechanisms, such as gravitational separation (creaming/sedimentation) because of differences between phase density, flocculation, coalescence, and Ostwald ripening. Droplet aggregation may occur due to coalescence or flocculation. The rate of Ostwald ripening increases with the increase in the water solubility of the oily phase, especially for polar oils, as plant-based antimicrobial agents. It is prevented by adding ripening inhibitors.

A post-pH-driven encapsulation widely applied to different polyphenols and food systems has been developed as a viable solution offering simplicity, speed, and environmental friendliness. It eliminates the need for heat, organic solvents, and complex equipment, and also contributes to reducing the food waste of plant-based food [283].

Recently, EOs are delivered through novel procedures such as electrospinning, nanoencapsulation, nanoemulsions, and nanocomposites to enhance antibacterial and antioxidant performance for better involvement with cell molecules [284,285]. Their functionality is explored to create a controlled release to a certain target or with a convenient release rate or under the action of selected external factors [286,287] to promote the predictability and reproducibility of release rates. Change in the concentration with time has practical significance in some situations, such as when it is desirable to have a higher concentration of BAC or drugs (antimicrobials or antioxidants) in the beginning to retard microbial and oxidative deterioration, while having a lower concentration when the product has reached the target. Fungicidal activity of essential oils (EOs) from various plants (*Salvia officinalis* (*Lamiaceae*), *Melaleuca alternifolia* (*Myrtaceae*), *Mentha arvensis* (*Lamiaceae*), *Baccharis dracunculifolia* (*Asteraceae*), *Baccharis uncinella* (*Asteraceae*), and *Cymbopogon nardus* (*Poaceae*)) has been tested in vitro by the control of mycotoxin-producing strains of *Aspergillus niger*, *Fusarium graminearum*, etc. Volatile EOs (*Eucalyptus camaldulensis*, *Cymbopogon schoenanthus*, and *Cymbopogon nardus*) affect the growth, sporulation, and mycotoxin production of *Aspergillus flavus*, *Aspergillus carbonarius*, and *Fusarium verticillioides* (aflatoxin B1, ochratoxin A, and fumonisin B1, respectively) and modify the expression of some toxin synthesis genes [288] depending on dose.

Nanoemulsion preparation can be achieved by two kinds of methods: (i) low-energy methods and (ii) high-energy methods. Low-energy methods produce small oil droplets with or without gentle stirring. They do not require specialized equipment but need high concentrations of synthetic surfactants, while in the case of high-energy methods, small oil droplet formation requires disruptive forces for oil and water phases produced by microfluidizers, high-pressure homogenizers, and sonicators [289,290].

Low-energy methods for nanoemulsion preparation are spontaneous emulsification (SE), phase inversion composition (PIC), phase inversion temperature (PIT), and emulsion inversion point (EIP) methods. The optimized type and concentration of surfactants and oils led to small oil droplets when the system composition or environmental conditions were convenient. The applications of nanoemulsions for the delivery of active functional additives include bioactive lipids, essential oils, flavor compounds, vitamins, phenolic compounds, carotenoids, etc. For each kind of active agent and application, the preparation method of the nanoemulsion and suitable conditions must be carefully selected to obtain the desired results. A few examples are mentioned as follows: Antimicrobial nanoemulsions of black pepper oil (4.3%) have been prepared by the PIT method by heating at 75 °C for 30 min in the presence of the non-ionic surfactant Tween 80 (9.7%) and then rapidly cooled to 5 °C for 15 min. The preparation of the nanoemulsions of a mixture of clove and cinnamon oil has been achieved by the EIP method, which was applied, while for the antimicrobial carvacrol oil or a mixture of cinnamon and coconut oils, and for the chitosan/citral oil nanoemulsions, the SE method was applied. It can be concluded that the nanoemulsions formulated using a low-energy method exhibited better antimicrobial activity against *Erwinia carotovora*, *Aspergillus niger*, and *Rhizopus stolonifer.*

In the sonication methods (as high-energy emulsification techniques), the samples are placed in a mixture of oil, water, and emulsifier. The disruptive forces created microbubbles because of the cavitation effects, and the high-intensity ultrasonic waves caused rapid pressure fluctuations. All these contribute to mixing the oil/water phases, converting large droplets into smaller ones [291]. The antimicrobial nanoemulsions from *Cleome viscosa* EO and Tween 80, chitosan/eugenol oil, which are effective against *Streptococcus pyogenes*, *S. aureus*, *E. coli*, *K. pneumoniae*, and *P. aeruginosa*, lemon myrtle and anise myrtle essential oils, geraniol and carvacrol, and thyme oil have been prepared by sonication. It has been established that the nanoemulsions amplified the antibacterial activity of EOs, as an increase in EOs’ ability to disrupt cell membrane integrity is promoted.

In the high-pressure homogenization (HPH) technique, a coarse emulsion is forced by a pump to pass through a small valve under intense shear, cavitation, and turbulent forces that break down the large droplets into small ones [291], as has been achieved for curcumin-loaded nanoemulsions. The black pepper oil nanoemulsions prepared by HPH show a good antimicrobial efficacy against bacterial pathogens, being useful in aquaculture, while the cinnamon oil nanoemulsions are applied as good antimicrobials against disease-related pathogens and food spoilage bacteria.

The microfluidizer uses a high-pressure pump to force a coarse emulsion of an antimicrobial carvacrol to pass through two separate very narrow channels and then makes them impinge on each other so that the fluids collide at high velocity, creating high shear forces and impact that disrupt the large oil droplets into smaller sizes [292]. The pump is a core component of the device, creating the consistent pressure needed to achieve the process’s powerful homogenization and cell disruption effects. Nanoemulsions have a longer shelf life, ranging from a few months to many years. Most of the ingredients useful for emulsification/encapsulation of BAC are also obtained from biomass.

Thermodynamically unstable micro-/nanoemulsion systems can be stabilized by using some ingredients such as the following:

(a) Emulsifiers of different provenances, such as plant-based (e.g., vicilin, whey protein isolate, soy or pea protein); polysaccharides, such as gum arabic or modified starch, pectin (sugar beet pectin), plant mucilages, octenyl succinic anhydride-modified starch; phospholipids, such as soy or sunflower lecithin; and natural (like quillaja saponin) or synthetic (Tween and Spans). Pea, lentil, and faba bean proteins have been used as effective emulsifiers for the formulation of 10 wt% algae nanoemulsions obtained by microfluidization. The best efficacy was shown by the lentil protein in terms of providing resistance to environmental stresses such as pH, ionic strength, and temperature changes, while the sesame protein is an effective emulsifier for forming fish oil nanoemulsions.
(b) Surfactants (sugar esters, polyoxyethylene) reduce the interfacial tension by electrostatic/steric stabilization.
(c) Various stabilizers, including ripening inhibitors (as corn oil, sunflower oil, medium-chain triglyceride oil), The accepted mechanism of stabilization in Pickering emulsions considers the formation of a steric barrier by solid particles adsorbing at the oil–water interface, particles being able to irreversibly attach to the oil–water interface, leading to a more efficient stabilization than surfactant adsorption.
(d) Texture modifiers as thickening and gelling agents (carboxymethyl cellulose, sodium alginate, or pectin) to thicken or gel the aqueous phase, thereby inhibiting droplet movement. Thickening agents increase the viscosity of aqueous solutions, whereas gelling agents provide semi-solid characteristics, forming a 3D network of crosslinked molecules.
(e) Weighting agents that inhibit gravitational separation, i.e., creaming and sedimentation, are hydrophobic substances such as brominated vegetable oil, ester gum, damar gum, and sucrose acetate isobutyrate.
(f) Interfacial stabilizers are important components for electrostatic deposition methods with modified polysaccharides (as chitosan hydrochloride electrostatic deposition coats oil droplets with polysaccharides), increasing the steric and electrostatic repulsion between them, inhibiting aggregation. Thymol nanoemulsions’ stability was increased by whey protein-coated oil droplets and chitosan hydrochloride [293].
(g) Ripening Inhibitors: Some antimicrobial nanoemulsions, especially those containing pure, polar, water-soluble EOs, are highly unstable due to a phenomenon known as Oswald ripening, by which small oil molecules form larger oil droplets, resulting in an increase in the mean droplet size over time, leading to creaming or coalescence, making the nanoemulsion unstable. By adding hydrophobic substances as a ripening inhibitor, like corn oil, sunflower oil, or medium-chain triglyceride oil, this instability is avoided for an optimum concentration. These little droplets help to increase the droplet’s surface area, which improves the functional qualities of the encapsulated component.
(h) Pickering emulsion utilizes solid particles as stabilizers, which accumulate at the interface between two immiscible liquids (typically as oil and water phases) and stabilize droplets against coalescence. Inorganic particles, including silica, clay, and hydroxyapatite, and also some organic particles, can effectively serve as Pickering emulsifiers [273,294]. Multiple Pickering emulsions (MPEs) can be excellent carriers of bioactive compounds, offering a robust background to design MPEs with functional and nutritional benefits. MPEs offer the following advantages: (i) reduced coalescence, bringing higher stability to emulsions; (ii) useful characteristics of many solid particles, such as conductivity, responsiveness, porosity, etc.; (iii) lower toxicity of some food-grade solid particles, leading to higher safety for in vivo usage.

Pickering emulsions are applied in many fields, such as biomedicine, food, fine chemical synthesis, cosmetics, etc. Formation of the oil-in-water (O/W) or water-in-oil (W/O) Pickering emulsion is determined by the wettability of solid particles at the oil–water interface. A three-phase contact angle θ (angle at the three-phase boundary of solid particles, continuous phase, and dispersed phase) characterizes such systems. O/W emulsions have θ < 90° (e.g., silica, clay), while W/O emulsions have θ > 90° (e.g., carbon black). Only when θ ≈ 90° do the particles effectively act as a Pickering stabilizer. Since particles tend to remain dispersed in either phase if they are too hydrophilic (low θ) or too hydrophobic (high θ). Amphiphilic particles are preferred, and they are obtained by modifying particles. Some biological and food-grade edible particles, such as starch, zein, soy protein, whey protein, and bacteria-related particles, are increasingly employed in the formulation of Pickering emulsions due to their excellent biocompatibility and biodegradability, as well as attractive potential applications in food and drug delivery fields.

(i) Antioxidants (as quercetin or curcumin) reduce oxidation in nanoemulsions.

**Figure 3:**
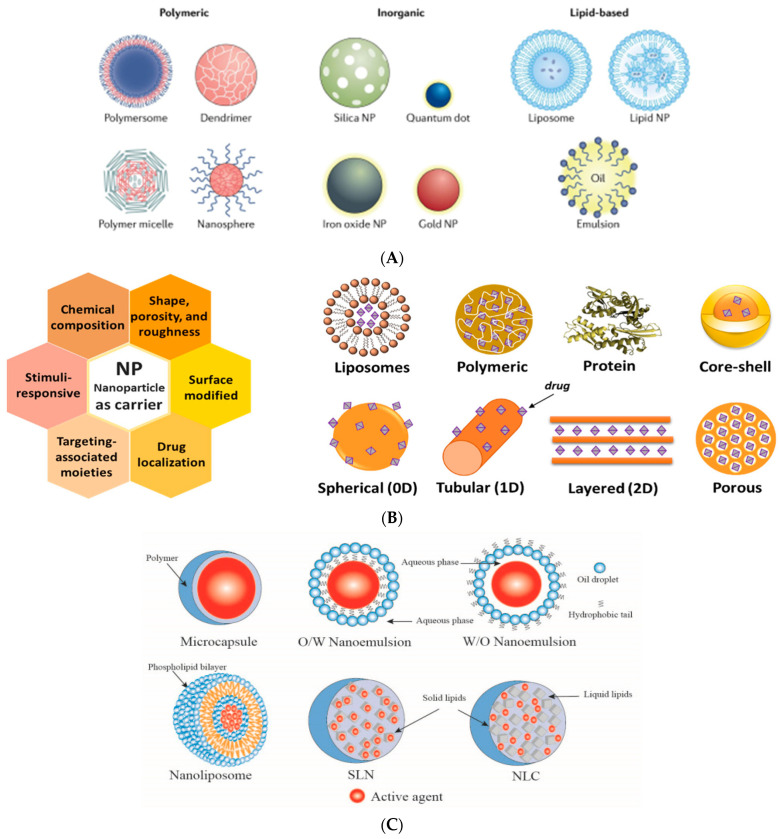
Variable morphologies of the emulsified/encapsulated bioactive additives by changes in chemical and physical parameters in the design of the nanoparticles (**A**), of different chemical natures, shapes, porosity, dimensionality (**B**), or using different carriers (**C**), adapted from Refs. [295,297,298]. (W/O; O/W) water-in-oil emulsions or inverse; SLN—solid lipid nanoparticles and NLC—nanostructured lipid carriers.

Emulsified/encapsulated (B)AC with different morphologies/structures/sizes, especially for those of nanosized scale, such as aligned, core–shell, and porous structures, which could be modified by adjusting devices [295,296,297,298,299] and the process parameters or by the selection of encapsulating materials or (B)AC possessing different activities, such as antimicrobial, antioxidation, ultraviolet protection, and pH responsiveness (Figure 3), have been developed. Nanoparticles of different chemical natures, shapes, porosity, dimensionality, etc. (Figure 3A), variable chemical and physical parameters are considered in the design of the nanoparticles (Figure 3B) or the use of different carriers (Figure 3C), etc. In the case of the polymeric NPs, a precise control of their characteristics is assured by control of hydrophilic/hydrophobic content. Surface modification is easy, but aggregation and toxicity are possible and have to be studied. Inorganic NPs offer unique electrical, magnetic, and optical properties, but many are insoluble and may have variable size, structure, geometry, and toxicity. Lipid-based NPs exhibit flexibility, high bioavailability, low encapsulation efficiency, and various physico-chemical properties.

#### 4.2.3. Micro and/or Nanoencapsulation

Micro- and/or nanoencapsulation are favorable alternatives to preserve and protect bioactive compounds/additives against inappropriate environmental circumstances and increase their bioavailability and stability, thus advising their applications in agriculture, food, and pharmaceutical industries. Nanoencapsulation and delivery systems enhance the stability and bioavailability of bioactive compounds. Nanoencapsulated additives are costly and need to be balanced by the potential benefits.

Polyphenols (proanthocyanidins, phenolic acids, and flavonoids, especially anthocyanins) with specific flavor profiles are highly unstable under processing and storage conditions. Two main alternatives of the encapsulation have been developed to protect the properties of the active compounds during processing, and applications include both physical and chemical ones. (i) The macro-, micro-, and/or nanoencapsulation by the incorporation into a suitable polymer matrix (of mainly biopolymers such as modified starch, chitosan, cellulose, carboxymethylcellulose, pectin, sodium alginate, gum Arabic, xanthan gum, pullulan, shellac, zein, maltodextrin, whey protein, galactomannan, polycaprolactone, sodium caseinate, etc.). (ii) Enzyme encapsulation and/or immobilization as novel technologies to improve the stability (stabilization) of the natural compounds, including flavors and fragrances.

*Encapsulation* has a number of benefits, including the following:

- Extended shelf life.
- Improved stability during processing and in the final product.
- Change in the structure from liquid to solid.
- Liquidity, dispersibility, and dosage accuracy in the final product are improved.
- Controlled release of aroma compounds, prolonging exposure to odor or taste; masking of taste and odor.
- Protection from external factors, separation of chemically unstable and highly volatile substances from environmental factors, protection from UV radiation, and degradation reactions under heat, oxidation, and dehydration.
- Improved safety by reducing the flammability of volatile substances [298].

Innovative strategies like nanoencapsulation, responsive hydrogels, and AI-driven optimization have significantly progressed [299].

Encapsulation alternatives are applied by the following methods:

(a) Physical-mechanical processes (e.g., spray drying, dripping, fluid bed coating, spray cooling/chilling, extrusion, emulsification and freeze drying, supercritical fluid or anti-solvent drying, co-crystallization, lyophilization, pan-coating, spinning discs deposition onto substrate, electrospinning, and copigmentation). Spray drying converts a nanoemulsion into a fine powder, which is easy to handle, store, and transport and is much more stable. Nanoemulsions in a fluid state are turned into powders having high encapsulation efficiencies (95–98%).
(b) Chemical processes (e.g., liposomes, coacervation or molecular inclusion in cyclodextrins, complexation, yeast encapsulation, emulsions, emulsion polymerization, interfacial polymerization, interfacial crosslinking), ionic gelation.
(c) Physico-chemical methods such as coacervation, sol–gel synthesis method, organic phase separation, solvent evaporation, layer-by-layer adsorption, and liposome entrapment.

Probiotic encapsulation in food science has had a rapid development, with new approaches such as 3D printing, spray drying, microfluidics, and cryomilling. Co-encapsulation of probiotics with bioactives presents a cost-effective and successful approach to deliver probiotic components to specific colon areas, improving viability and bioactivity. Electrohydrodynamic techniques generate fibers with diverse properties that protect the active/bioactive additives from a harsh environment. These procedures are adaptable, uncomplicated, and easily scalable; the products that result have good quality and functionality across various domains, including food [300].

Freeze drying is considered a standard method for encapsulation, being useful to obtain high-value food products [301].

Polysaccharides as carriers can be used as color and flavor enhancers, to prolong the shelf life of products since they can prevent oxidative degradation, and also as target cancer cells, alone or in combination with the standard chemotherapeutic. Retention and stability of the flavors of the products are strongly related to the consumers’ acceptability of the products, and a good formulation of delivery systems of flavor compounds is necessary. Flavor is a product quality parameter that is very complex and very difficult to control. The different nonvolatile constituents (e.g., proteins, lipids, carbohydrates, salts, etc.) show a great impact on the retention of the flavor compounds. Different degradable polymers (whey proteins, chickpea protein, gums, pectins or gelatins, gum Arabic, chitosan, sodium alginate, xanthan gum, pullulan, modified starch, cellulose and carboxymethylcellulose, shellac, zein, maltodextrin, whey protein, galactomannan, polycaprolactone, and sodium caseinate) have been tested as encapsulating materials for the bioactives. Homogenization, probe sonication, electrospinning, and 3D printing have been utilized by entrapment/encapsulation, resulting in nanoparticles, nanocapsules, nanofibers, films, nanoemulsions, 3D printed composites, and hydrogels [302,303]. The main encapsulated BACs in the food area are vitamins, essential oils, plant extracts, caffeine, bioactive peptides, fatty acids, flavonoids, carotenoids, and terpenes. Encapsulation can assure the stability of formulated systems, increase the release time, or determine the controlled release of BAC, retain and protect active properties, reduce lipid oxidation, and maintain organoleptic properties and bioactivities even in extreme thermal, radiation, and pH conditions [304]. Chitosan NPs had a greater impact on extending the shelf life in comparison with chitosan alone [305].

Dietary fibers possess specific technological and functional attributes that assure their use as carriers for bioactive and volatile compounds, thickeners, oil texturizers, moisture retention agents for the preparation of different types of products, etc.

Interactions of (bio)active additives and biopolymers through hydrogen bonding, Van der Waals forces, non-polar hydrophobic interactions, cross-linking, and weak electrostatic interactions are responsible for the formulation of efficient and successful delivery systems. Oligopeptides, enzymes, and other macromolecules are immobilized via covalent bonds to the surface or simple electrostatic adsorption. These interactions depend both on the properties and compositions of the components of the system and also on the production conditions, such as temperature, time, pH, ionic strength, etc. Hot melt extrusion technology applied in the field of the nutraceutical industry has been successfully adopted to prepare solid molecular dispersion of active pharmaceutical ingredients. The separation of the insoluble complex coacervates and the liquid is assured by electrostatic binding between oppositely charged polymers [306].

Micro-encapsulation of active ingredients is an efficient, cost-effective, and environmentally friendly method. Biodegradable microcapsules and controlled release of encapsulated active agents are two innovative directions very interesting to study and have applications in various fields, such as agriculture, pharmaceuticals, cosmetics, the textile industry, and others [307]. For agricultural areas, the encapsulation is efficient for the formulation of biofungicides, biopesticides, bioherbicides, and biofertilizers, and this also includes encapsulated essential oils, lipids, phytotoxins, medicines, vaccines, microbial metabolites, etc. By these studies, the stability of formulated systems has been improved, the release time increases, the active properties are retained, lipid oxidation is reduced, organoleptic properties are maintained, and the bioactivities are kept even in extreme thermal, radiation, and pH conditions. By encapsulation of plant-based preservatives, their handling is improved, and ease of use, as well as their potency, is enhanced. Encapsulation of volatile compounds into inorganic nanocarriers, such as nanoclays, prolongs the release of active additives and protects them from harsh processing conditions. As an example, the BAC (as curcumin or thymol) is dissolved in a suitable solvent by sonication, and then the nanocarrier is added. The loaded nanocarriers are separated by centrifugation, washed, and vacuum-dried at room temperature. The hydrophobic BACs (curcumin)-loaded nanocarriers are coated with a PEG-6000 polymer. Mesoporous silica nanocarriers load large amounts of natural BACs (up to 500 mg/g). The loaded nanocarriers are shelf-stable, have a very slow initial release, and after 2 months have about 50% retention of the encapsulated compounds [308,309].

The approved nanoparticles by EU Commission Regulation No. 10/2011 are silica, carbon black, titanium nitride, and silicic acid. The design of the nanocarrier highly influences the loading degree, the retention, and the subsequent release. Porous silica nanoparticles are the most suitable carriers for potential food packaging applications, having design options for the pore structure, surface area, and surface chemistry. The encapsulated compounds have applications in the biomedical field for drug delivery, diagnostic imaging, and the stabilization of bioactive proteins [308].

#### 4.2.4. Protein–Polysaccharide Nanocomplexes

Protein–polysaccharide nanocomplexes offer a stable encapsulation structure for various vitamins, ensuring sustained release. Colloidal and molecular interactions drive nanocomplexation behavior, with or without chemical covalent bond formation. They disrupt bacterial cell membranes, inhibit cellular respiration, and interfere with bacterial replication, effectively preventing the growth of pathogens, so they are useful for extending the shelf life of perishable food products [310].

#### 4.2.5. Electrospinning/Electrospraying

Electrospinning/electrospraying as an encapsulation method is characterized by cost-effectiveness, versatility, and scalability. Installation setup consists of a high-voltage power source connected to a metallic nozzle and a metallic collector. To an ejected drop of a polymer solution at the tip of the nozzle is applied a high voltage. The electrostatic repulsive force acts on the drop surface and counteracts the surface tension, and a solution jet is ejected and deposited onto the collector. Solutions, emulsions, or suspensions are turned into small droplets that are exposed to an electric field, and the tiny droplets are dried to solidify into either nano- or micro-sized particles as a nanofiber mesh. The electrospun nanofibers and/or electrosprayed nanoparticles are obtained, and they protect the encapsulated BAC [311,312,313]. Electrospinning/electrospraying is a one-step process operating at ambient conditions (atmospheric pressure and room temperature) and produces dried micro-/nanostructures [314]. 

New solutions have also been designed to diversify the applications for the natural antimicrobial and antioxidant agents’ applications [315,316,317,318,319].

The biological functions of the metal-based nanoparticle (MBNP) electrospun nanofiber are mainly designed for new tissue engineering materials due to their excellent antibacterial, anti-inflammatory, and wound-healing abilities. Such nanofibers have a core–shell morphology similar to the porous arrangement of extracellular matrix (ECM), so they are favorable for tissue regeneration. The loaded MBNPs are wrapped and fixed and can be controlled to release in situ at the wound site. MBNPs are also widely used in drug delivery, biological imaging, and cancer treatment. The most commonly used NPs applied in this field are AgNPs, AuNPs, and CuNPs. The excellent antibacterial and anti-inflammatory properties of the AgNPs help to prevent the wound deterioration; the AuNPs promote tissue regeneration by their anti-inflammatory and antioxidant effects, while the CuNPs contribute to the reconstruction of the extracellular matrix (ECM). By filtration within the hepatobiliary and renal systems, the MBNPs can accumulate in the liver, spleen, and kidneys, these being the potential toxic side effects. In order to reduce such effects, these MBNPs and their integration with appropriate carriers are necessary [318]. Both biopolymers (e.g., collagen, gelatin, alginate, chitosan, hyaluronic acid and silk fibroin) and synthetic biodegradable polymers (such as polycaprolactone (PCL), polyvinyl alcohol (PVA), polyethylene oxide (PEO), polylactic acid (PLA), polyglycolic acid (PGA), polyglycerol sebacate and polyurethanes) have been electrospun into nanofibers, and also biofunctionalization of polymers has been applied to improve cellular interaction and for a wide range of tissue type integrations, namely neural, vascular, cartilage, bone, dermal and cardiac.

New solutions have also been designed to diversify the applications for the natural antimicrobial and antioxidant agents’ applications as nanoparticles (NP) *incorporated*/*encapsulated into appropriate carriers* in packaging and other types of high-performance, eco-friendly materials. By combining eco-friendly activation processes, such as cold plasma or gamma irradiation exposure, *the surface functionalization* of the materials is achieved by covalent bonding of the bioactive plant oils (clove essential oil and rosehip seeds vegetal oil), with the antioxidant and antibacterial activities being improved and stable [315,316].

A growing interest in the development of micro- or nanoparticles loaded with active additives, such as polyphenols, carotenoids, fatty acids, phytosterols, probiotics, vitamins, minerals, and bioactive peptides, to deliver them for various applications is evident both in research and industry, driven by the benefits of encapsulation. The Technology Readiness Level (TRL) for most of the encapsulation methods is going beyond TRL 6. The controlled release of the encapsulated materials at a specific time and target in the human body, and their use directly in the food processing systems, is also possible [317].

## 5. Green Synthesis of the Nanosized Active Additives

At the beginning of the 20th century, many methods have been employed to reduce the chemical use in the production/synthesis of the NPs known as *top-down methods* (e.g., physical or mechanical methods like grinding and ball milling applied to the bigger metal particles to turn them into the nanometer scale range) and *bottom-up* methods (e.g., chemical methods and biological methods, in which some chemicals are involved) [319]. The developing green methods for the synthesis of NPs are based on the biogenic route utilizing natural extracts from plant parts (leaves, roots, flowers, fruit, plant waste), microbial extracts, and biometabolites from organisms like bacteria, algae, fungi, etc. Compared to conventional synthesis, green nanoparticle fabrication is an environmentally friendly, toxic-free procedure, envisaging also waste management, which allows a reduction in energy consumption of 30%, cost savings of up to 40%, and an increase of 50% of production output and waste management [320]. The biosynthesized NPs loaded into various matrices impart those suitable mechanical and barrier properties for applications [321]. The biological materials are effective oxidizing and reducing agents and are rich sources of chemicals and phytochemicals. There are three types of green synthesis methods, namely those using (a) microorganisms, (b) direct biomolecule templates, and (c) plant extracts.

The microorganisms of algae, actinomycetes, fungi, prokaryotic bacteria, and yeast, by their intracellular or extracellular activities, generate certain enzymes which, alone or with other organic molecules with anionic functional groups, are present around the cell wall and found on the surface of the microorganisms, being responsible for the NPs production. The Gram-positive bacteria (such as *S. aureus* and *Bacillus cereus*), which are composed of peptidoglycan, teichoic acids, lipoteichoic acids, polysaccharides, and proteins, have been used in NP production [322,323]. Mobilization and immobilization of metals, in some cases, are activated by bacteria, which can reduce metal ions and precipitate metals at the nanometer scale. The enzymatic action of the molecules existing on the surface of the fungal cells is widely responsible for the NPs synthesis. Among the versatile fungal species useful for the synthesis of various metal NPs (like Ag, Au, and Zn) and metal oxide NPs (such as AgO, ZnO, CuO, TiO_2_, etc.), the following can be mentioned: *Aspergillus terreus*, *Aspergillus flavus*, *Bryophilous rhizoctoni*, *Pleurotus ostreatus*, *Candida albicans*, etc. *Yeast* and *actinomycetes* have also been exploited for the NPs synthesis. The mycofabrication processes, involving fungi in the extracellular or intracellular syntheses of metal nanoparticles, can be easily scaled up, streamlined downstream, handled economically, etc. Fungal functionalized nanostructures enhance the food packaging functionality, food processing, and safety [324]. Very effective biotemplates in the metal and metallic oxide NP syntheses also are DNA, nucleic acids, viruses, and some biochemicals, such as glucose or sugar and citric acid.

*Plant-based syntheses* (*phytonanotechnology*) can be intracellular, extracellular, and phytochemical. Plant phytochemicals can both reduce and stabilize nanoparticles. The solubility can be increased with the use of a complexing or chelating agent in the salt solution [325]. In order to obtain the metal ions in the nanometer range by the intracellular methodology, the plants are grown under metal salts at an appropriate concentration. The extracellular approach is based on the ability of the phytochemicals to reduce the metals, while the biochemicals act as both the capping and stabilizing agent. Plant-mediated synthesis of nanoparticles is preferred over chemical synthesis due to its eco-friendliness, non-toxic by-products, simplicity, extensive antimicrobial activity, etc. The phytochemical-mediated synthesis by organic acids in composition is used to stabilize iron oxide nanoparticles, which are high in magnetization, non-toxic, biocompatible, and with applications for drug delivery [326].

*Silver nanoparticles* (Ag NPs or nanosilver) show antimicrobial and antifungal activity, and it has been demonstrated that they are a good biocide against bacterial strains, *algae*, *fungi*, *and possibly some viruses* [327], so they have a wide range of applications, including biomedical, environmental, and agricultural, from disinfecting medical devices and home appliances to water treatments. Several eco-friendly processes have been reported for green synthesis of AgNPs, using aqueous extracts of whole plants or their parts, such as the leaves, roots, bark, flowers, fruits, seeds, rhizomes, peels, agricultural waste, fungi, etc. Microorganisms such as filamentous fungi, bacteria, lichens, algae, higher plants, possibly some viruses, and microbes have also been used. AgNPs are produced by oxidation of Ag+ to Ag^0^ by different biomolecules from the plant extracts. A green method using *Citrus X sinensis* fruit extract and *Chlorella vulgaris* aqueous extract, with the assessment of their catalytic reduction, has been developed [328,329]. The phytochemical type and their concentrations, extraction type and solvent, temperature, pH, and reaction time determine the size, shape, and stability of the produced AgNPs. AgNPs as antimicrobial activity varies with NP morphology as spherical AgNPs > disk-like AgNPs > triangular AgNPs. There are some efficient systems for green synthesis of AgNPs, as silver nitrate is used as a metallic precursor and the *Moringa oleifera* leaf extract with different concentrations as a reducing as well as a capping agent, or the *Eucalyptus camaldulensis* and *Terminalia arjuna* extracts, as well as their combinations, as reducing and capping agents exhibited strong potential in rapid reduction of silver ions for the synthesis of AgNPs [330]. Biologically active Ag-NPs from the *Lavandula mairei* Humbert (*L. mairei*) plant exhibited high antibacterial activity against ESBL-producing multidrug-resistant (MDR) strains and also against carbapenem-resistant and non-carbapenem-resistant strains of *A. baumannii*, as well as very interesting antioxidant activity [331]. The extract of Cocculus Pendulus as a reducing and stabilizing agent is extremely poisonous to Gram-positive bacteria and is a promising antibiotic against bacterial infections [332]. AgNPs synthesized and coated by different leaf extracts under the acceleration of ultrasound possess various shapes, sizes, and even bioactive properties, substantially depending on the kind of extracts [333]. Olive leaf extract has been used as the reducing and capping agent for AgNO_3_ to obtain stable, spherical AgNPs with a size of 10.6  ±  2.5 nm. AgNPs used in biological applications have been obtained by means of an aqueous two-phase system with citrate as a reducing agent [334]. Other interesting methods have been elaborated [335].

Accumulation of AgNP in the human body or the environment, particularly through food packaging waste, raises concerns about long-term toxicity. The use of nanosilver is controversial, as are other leachable antimicrobial agents, as substances embedded into plastic articles that release antimicrobial features via diffusing or leaching, thereby stopping microbial growth in the surrounding environment. Leaching will cause the depletion of the agent, especially in applications that come in contact with water. To avoid the potential toxicity, the concentration and controlled release of AgNPs must be carefully regulated. The use of traditional Ag-based antibacterial agents occurs by uncontrollable silver release, so it is difficult to make a correlation between antibacterial performance and biosafety. The regulation of silver release behavior with excellent biocompatibility is applied for an efficient treatment of drug-resistant bacterial-infected wounds [336].

*Gold nanoparticles* (AuNPs) have been obtained by the reduction in chloroauric acid (HAuCl_4_) using *Acai berry* (AB) and *Elderberry* (EB) extracts [337] or the aqueous extract of *Zingiber officinale* rhizome. AuNPs with particle sizes ranging from 2.5 nm to 90 nm were synthesized in situ using chitin nanogels by varying the reaction temperature and chloroauric acid concentration, while novel biocompatible chiral AuNPs with a diameter of 54.4 ± 14.9 nm with potential application for biosensing were prepared by an in situ reduction of HAuCl_4_ with alginates and a chiral-inducing and stabilizing agent [338].

*ZnO NPs* possess unique semiconducting, piezoelectric, and optical features, biodegradability, and low toxicity and are low cost, easy to synthesize, non-toxic, and biocompatible. High-surface-to-volume ratio and direct contact with the bacterial cell wall of the ZnO NPs explain the better destruction of bacterial cells than their micro- or macroscale equivalents. The ZnO surface has OH groups; therefore, it is easily functionalized. ZnO NPs are easily absorbed by the body when they are utilized as food additives. ZnO-NPs enhance the growth and yield of food crops. As examples in their presence, peanut seeds increase the rate of growth and development of peanut roots and stems, improve the growth rates of cucumbers, the growth rate of mung beans and chickpeas, and mediate photosynthesis of wheat and maize at low concentrations, etc. Potential therapeutic biological activities/applications of ZnO-NPs are powerful antioxidants, useful in an important additional treatment for chemotherapeutic drugs that induce male reproductive failure, anti-diabetic, antibacterial, fungicidal, and anti-inflammatory for microbial infection and inflammation, and also they can be used as drug carriers, imaging agents, and biosensors [339,340].

ZnO NPs have been synthesized by conventional methods like chemical reduction, laser ablation, solvothermal, inert gas condensation, and the sol–gel method, which require some toxic chemicals, high pressure, laser radiation, and inert gases like helium, which are expensive. The green techniques for the synthesis of ZnO NPs have been developed using plants, fungi, bacteria, and algae as aqueous extracts of sea lavender, *Limonium pruinosum* (L.) *Chaz*., as a reducing, capping, and stabilizing agent [341] or lychee peel [342]. ZnO NPs were also prepared using leaf extract of *Ocimum tenuiflorum* (holy basil, tulsi) or aqueous extracts of *Carica papaya* leaves, which reduce the zinc acetate to zinc oxide nanoparticles and act as surface stabilizing substances for zinc oxide nanoparticle production. Drop-wise addition of the nettle extract (10% *v*/*v*) has also been used to obtain the synthesis of ZnO nanoparticles with the aqueous extracts of *Carica papaya* leaves, *Aloe vera* leaf extract, or *Jatropha podagrica* leaf extract [343]. Green-synthesized ZnO nanoparticles were found to be excellent antioxidant, antibacterial, biocompatible, and effective dye degradation nanomaterials.

Spherical green synthesized ZnO NPs are of great importance in treating clinical pathogens such as *Staphylococcus aureus* and *Enterococcus faecalis* because they are biosafe and biocompatible and suitable for therapeutic purposes, with promising useful medicinal applications, as in the treatment of numerous human infectious pathogens. Brown algae are good sources of alginate and are used as a reducing agent in the synthesis of nanoparticles. ZnO-NPs have been synthesized by using the brown alga *Fucus vesiculosus* and also to extract alginate from the same algae [344]. Some bacteria (*Bacillus megaterium*, *Sphingobacterium thalpophilum*, *Staphylococcus aureus*, *Halomonas elongata*, *Candida albicans*, and *Aspergillus niger*) can produce ZnO-NPs utilizing specific enzymes. The 61 nm and 25 nm ZnO-NPs produced by fungi can be employed as antibacterial agents, while egg albumin and gelatin have been tested for the synthesis of ZnO-NPs with antibacterial and anti-angiogenic properties [345].

Innovative combinations of various nanoparticles as nanocontainers and nanocarriers, such as silver, zinc, halloysite nanotubes, and polymer combinations, have also been proposed in developing metallic implant/drug/device combinations for controlled drug release in dental and orthopedic applications, exploiting synergistic effects [346]. The leaf extract of the *Ocimum tenuiflorum* plant has been utilized in the synthesis of Cu, Au, and AgNPs. Biosynthesized ZnO and CuO NPs from *Urtica dioica* leaf extract were used as antimicrobial actives in chitosan films, and they also exhibit antimicrobial properties against both Gram-positive and Gram-negative bacteria.

An efficient and ‘green’ method of coating the electrospun polylactic acid (PLA) nanofiber composite using Ag- and Zn-coated halloysite nanotubes (HNTs) has antimicrobial properties different from metal-coated HNTs, polymer combinations, and metallic implant drug/device combinations, being interesting for controlled drug release in dental and orthopedic applications. CuNPs were obtained using green tea leaf extracts, while black tea leaves were used to prepare Cu-Fe bimetallic NPs useful for the removal of drugs from contaminated water. The preservation of strawberries has been improved by using a novel coating formulation of chitosan and iron oxide nanoparticles in the presence of ginger and garlic extracts, which assures the green synthesis of Fe_3_O_4_ NPs combined with varying concentrations of 1–3%.

*MgO NPs* are widely used to prepare nanocomposites for applications in antibacterial food packaging.

The use of *zero-valent iron* nanoparticles for the delivery of metal-containing foods via encapsulation or binding to biopolymers is a topic of recent studies. Iron deficiency is a widespread nutritional problem affecting millions of people, leading to various health issues, including anemia. For iron fortification of meat and meat products, ferrous sulfate and ferric pyrophosphate have been tested, and they have a good bioavailability and minimum impact on the sensory and nutritional qualities of meat products. Technological challenges and solutions include encapsulation, chelation, and microencapsulation techniques. Food-grade gelatin was used to encapsulate iron nanoparticles [346,347,348].

*TiO_2_ NPs* have been widely used for active food packaging, photocatalysis, and antimicrobial applications due to their excellent stability and antibacterial properties, mainly against Gram-positive and Gram-negative bacteria. It was demonstrated that the low migration rate of titanium ions is insufficient to cause harm to human health; thus, they can be considered a safe additive for food applications. TiO_2_ nanoparticles and essential oils act as active ingredients in polymer materials with strong antibacterial and antioxidant activity. Plants, microorganisms like fungi and bacteria, *Cannabis sativa* leaf extract, and *Impatiens rothii* Hook. f. leaf (IL) extract as a capping and reducing agent are used for the synthesis of TiO_2_ NPs [349]. Biologically mediated TiO_2_ nanostructures can be integrated on feasible platforms for industrial applications.

*Biopolymer NPs.*

Microcrystalline cellulose, bacterial nanocellulose (BNC), and cellulose nanocrystals have been produced through biological valorization of lignocellulosic agricultural and industrial waste. Bacterial cellulose, or bacterial nanocellulose, can be synthesized in the presence of multiple bacterial species, such as *Acetobacter* sp. and *Gluconacetobacter* sp. [350,351]. *Nanostructured cellulose (NC)* is a sustainable biomaterial for diverse biotechnological applications; however, its production requires hazardous chemicals that render the process ecologically unfriendly. New resources and methodologies have been proposed. Sustainable and green NC production is based on the combination of mechanical and enzymatic approaches [352]. Cellulose nanofibrils (CNFs) are attractive because of their low cost and abundance of lignocellulosic biomass from which they can be extracted, non-toxicity, and biodegradability. To extract CNFs from biomass through fibrillation requires high energy; therefore, the sustainability is reduced. Developed mechanical, chemical, and enzymatic pretreatments before fibrillation reduce the energy requirements of CNF extraction and/or impart functionality in resulting nanofibrils, or new procedures are proposed [353]. Polydopamine (PDA) imparts multifunctionalities onto cellulose nanofiber (CNF) films through nanocoating, enhancing the properties of the films, such as Young’s modulus, dielectric, and antioxidant activity [354]. From the Colombian cocoa pod husk, cellulose microfibers have been isolated by the extraction via chemical treatment and pressure [355].

*Starch NPs*: Nano starch syntheses could occur by enzyme hydrolysis, electrospinning, electrospraying, nanoprecipitation, self-assembly, cold plasma treatment, ball milling, ultrasonication, and high-pressure homogenization as environmentally friendly methods. Starch NPs have special qualities like being biodegradable, biocompatible, and having better barrier properties [356].

*Chitosan NPs* have been synthesized both for different purposes and by using various methods, such as ionotropic gelation, polyelectrolyte complexation, emulsification, solvent diffusion, microemulsion, reverse micelle formation, electrospinning, etc., and have different structures, shapes, and sizes [357]. The extraction of chitosan from fungal organisms is simple and environmentally friendly and uses in situ green science principles, and subsequent conversion into nanoscale materials as chitosan nanoparticles and nanocomposites has been developed [358]. Chitosan nanomaterials are useful as antimicrobial, anticancer, and agro-active agents. The ethanol extract of *Martynia annua* L. contains more pharmaceutically valuable phytochemicals than other solvent extracts. The resulting chitosan nanoparticles from a green synthesis using this extract showed effective antibacterial activities to control microbial pathogens. The antibacterial activity varies in the order of *Bacteroides fragilis* > *Streptococcus oralis* > *Propionibacterium acnes* > *Pseudomonas aeruginosa* > *Staphylococcus aureus* > *E. coli* > *Bacillus cereus* > *Streptococcus mutans* > *Aeromonas hydrophila* > *Streptococcus faecalis.*

*Nanoalginates*, as particles or fibers, are an advanced category of alginate materials. They are not practically useful without the incorporation of some other polymer or binary components (polymers, surfactants, solvents).

*Lignocellulosic NPs synthesis:* An eco-friendly process for producing LCNFs (lignin contents between 14 and 22 wt% and cellulose contents of 50–73 wt%) offers a scalable pathway for developing high-performance nanofibers with broad industrial applications and has been developed starting from rambutan peel as the biomass source [359].

Green syntheses of various NPs have been reviewed in several papers and books, so only the most recent are given here [360], evidencing their developments, advantages, impressive activities, efficiencies, and roles of NPs. The role of specific phytochemicals (phenolic compounds, terpenoids, and proteins) in plant-mediated nanoparticle synthesis has been emphasized, together with their influence on particle size, stability, and properties. The potential applications of these bio-derived nanoparticles, particularly with regard to drug delivery, disease management, agriculture, bioremediation, and applications in other industries, have been demonstrated. Bio-inspired nanoparticles derived from plants exhibit intriguing pharmacological properties, making them highly promising for use in medical applications due to their biocompatibility, bioavailability, and nano-dimensions. Polymers derived from natural biomass have emerged as a valuable resource in the field of biomedicine due to their versatility, offering several advantages, including biological effects, biodegradability, biocompatibility, and non-toxicity. Polysaccharides (chitosan, starch, cellulose, etc.), peptides, proteins (gelatin, collagen, and albumin), and lignin have demonstrated promising results in various applications, including drug delivery design [361,362]. NPs and encapsulated therapeutic molecules into polymeric NPs are extensively utilized in biomedical applications for sustained drug release, increasing the half-life of actives, improving drug efficacy and safety, reducing unwanted side effects, and enhancing the acceptance and compliance of patients. The recent developments of NPs for different biomedical applications include the treatment of inflammation, cancer, and infectious diseases; implants; prosthetics; and theranostic devices. The NPs composed of natural and synthetic biomaterials have found application in disease diagnosis. In tissue engineering and regenerative medicine, polymeric nanofibers are used in neural, vascular, cartilage, bone, dermal, and cardiac tissues. NP functionalization with molecules capable of improving receptor-mediated endocytosis has been explored for the treatment of several brain disorders, such as Alzheimer’s and Parkinson’s diseases, brain cancers, ischemic stroke, and epilepsy.

It can be concluded that the green syntheses of NPs utilize biological sources like plant extracts as reducing and stabilizing agents, microbial synthesis by bacteria, fungi, and algae, and enzymes to catalyze nanoparticle formation, avoiding the harsh, toxic chemicals and high energy requirements of traditional methods, reducing pollution, and reducing waste. Biological sources are readily available and inexpensive, making the process more economical and safe for humans and minimizing adverse environmental effects, being developed according to the sustainability principles. They are eco-friendly approaches with potential for large-scale applications in various fields like biomedicine, environmental remediation, photocatalysis, and energy, and they are also cost-effective, making them suitable for industrial applications. Olive oil waste (OOW) is used as a source for nanoparticle synthesis, particularly in the Mediterranean region. The practical applications of OOW-derived NPs are in water purification, pollutant degradation, drug delivery systems, antimicrobial activity, and cancer therapy [346]. Green synthesized nanoparticles are also very active ingredients acting as catalysts and antimicrobial agents and offering new possibilities in health care; they can be incorporated into cosmetics for UV protection and into textiles for antimicrobial and self-cleaning properties. In agriculture, they can be used to enhance crop yield, improve soil health, and control pests. Ensuring consistent, reproducible nanoparticle properties across different batches is crucial for large-scale applications. Toxicity assessment and standardized protocols of synthesized nanoparticles are essential to ensure quality, reproducibility, and comparability, especially for biomedical applications. The European Food Safety Authority (EFSA) and the U.S. Food and Drug Administration (FDA) standardize approval processes by establishing uniform scientific criteria, risk assessment methodologies, requirements for food products and ingredients/additives, ensuring safety, etc.

*Challenges:* It is necessary to mention some concerns related to NPs’ toxicity, dose dependence, reproducibility, etc. (see below).

## 6. Controlled/Targeting Release of (Bio)Active Agents

A technical system that comprehensively controls the spatial and temporal dosage distribution of (bio)actives in the environment/body is named a controlled delivery system. Generally, the term “controlled release” (CR) indicates the delivery systems with optimal control over release rate and techno-functional performance under external factors such as temperature, pH, medium, ultrasound, light, oxidation/reduction potential, magnetic or electrical fields, mechanical triggers, etc. [363,364,365]. A detailed control of the delivery systems’ characteristics and external factors, both in the human body, food products, environment, and other materials, is necessary. The Controlled-Release Packaging (CRP) concept defines a package having a modified concentration of an active compound inside it, developed in order to prevent/retard the deterioration of the food and to extend its shelf life. Controlled release of active compounds and new strategies in drug release and targeting are monitored in biomedical fields, such as tissue engineering and others, to establish a suitable concentration of drug to meet treatment needs. This is necessary, as an uncontrolled drug release could lead to high toxic concentrations initially or then to ineffective concentrations of the drugs. Generally, the release process for a bioactive with a particular concentration versus time pattern follows a certain release profile, which is described by a burst release/effect at the beginning of the process as a rapid release (in a short time) of a high quantity of the BAC. This is followed by either a sustained/prolonged release of the (bio)active with a fixed rate, which is relatively constant. In some cases for therapeutic purposes, a delayed, triggered or targeted release (release of a bioactive at or near the designed physiologic site of action), etc., is applied. Its concentration changes with time, following a “concentration profile” at different controlled rates. The active additive release (antimicrobials, antioxidants, flavors, aromas, etc.) in a controlled manner is designed to improve safety during extended storage and consumption. The kinetics and mechanism of controlled release are necessary to be established as the main objectives necessary to apply a CR for active agents [188]. The food and packaging interaction could occur by migration, permeation, and sorption. Migration of contaminants, plasticizers, thermal or UV stabilizers, adhesives, inks of a printed packaging surface, slip additives, solvents, monomers and oligomers, dioxins, nitrosamines from plastic polymers, or food additives that could enhance quality are important aspects necessary to be studied in detail. There are several types of migration, such as overall migration and specific migration; three categories related to food systems: non-migrating, volatile, and leaching systems; and also migration based on coefficient of diffusion, contact migration, gas-phase migration, penetration migration, and set-off migration related to the mass transfer of inks, varnishes, and coatings from the outer printed side to the inner side of the packaging films by stacking (e.g., of printed cartons) or during reeling (e.g., winding printed wrappers into a reel) that could be either visible or invisible depending on the specific substance and condensation/distillation migration.

Various types of controlled release systems and mechanisms for encapsulated active additives are known, depending on the main factors that control them (Table 4).

**Table 4:** Types of controlled release mechanisms and some factors affecting them [363,364,365,366,367].

No	Type of CR Mechanism	Some Characteristics and Dependence on Main Factors
1.	Dissolution	Easiest to occur; thermodynamically compatible
2.	Diffusion	The concentration gradient is one main factor influencing behavior
3.	Solvent-activated	Accompanied by swelling; chewing gum
4.	Osmotically-controlled	Similar to solvent-activated, the length of channels
5.	Barrier properties	Permeability; wall thickness
6.	pH-controlled	Targeted delivery; GIT delivery
7.	Temperature-activated	Glassy and rubbery; shrink/swelling
8.	Pressure-activated	Fragments; force fractured release
9.	Melting-activated	Physical state changes: Melting point
10.	Receptor delivery	Targeted release; ligand/receptor
11.	Erosion-controlled	Degradation is prevalent; no transport
12.	Enzyme-controlled	High sensitivity; smart carriers
13.	Nanosystem- controlled	Size and shape, microstructure, molecular interaction, density, environment, rigidity/flexibility, surface chemistry of the nanotextures, bacteria specificity (e.g., Gram-positive and Gram-negative), motility, etc.

The diffusion, dissolution, erosion, barrier properties, pH change, solvent swelling, receptor, enzyme control, degradation, fragmentation, pressure-, melting-, and temperature-activated microstructure, type of encapsulation material, molecular interactions, and environment affect the release rate of different BAC/drugs [363,364]. Controlled release in nanosystems is also dependent on the type of nano-/microstructure, particle structure, size, shape, molecular interactions, surface modification, environment, coating, surface charges, bioactive types, etc.

The highly variable design of biopolymeric CR formulations regulates various release mechanisms/profiles. Different biopolymeric nanocarriers, including nanoparticles (nanospheres/nanocapsules), protein/polysaccharide complexes, nanolaminated coatings, and nanofibers, have been tested as CR systems [365,366,367]. Multiple-layer coatings of biopolymers are promising strategies for the CR of probiotics.

Antimicrobial agents (AMs) can be introduced into food packaging systems or into drug delivery systems by blending, immobilization, or coating, but unfortunately, BACs can migrate from them into products/drugs. Migration can be hindered by encapsulation, incorporation of antimicrobials in matrices, or chemical bonding. Four types of systems are proposed for the controlled release of the AM: (i) one-layer system for the AM combined or chemically attached to the packaging material; (ii) a two-layer system for an AM coated on the outer layer of the packaging material, or the antimicrobial matrix layer (outer layer) is covered with the inner/control layer; (iii) the headspace system, in which the volatile antimicrobial agent is introduced into the matrix and then released into the packaged food headspace; and (iv) a headspace system with a control layer for the permeation of volatile antimicrobial agents while maintaining a particular concentration in the headspace region. In the first two systems, the AM is released by a diffusion release mechanism, while for the last two systems, the evaporation process into the headspace controls the release of volatile AMs.

Mathematical modeling of CR is extensively studied in drug delivery, but many of the modeling methods are also applied to food. The established appropriate mathematical model helps to predict the formulation parameters suitable to achieve a specific release behavior, and designing new products could be optimized. Four main mechanisms are involved in releasing bioactive agents from the encapsulation matrix: diffusion through the food matrix, dissolution, swelling, and degradation or erosion. In nonbiodegradable polymers, the dominant mechanism is diffusion, but in biodegradable polymers, swelling and erosion are also involved (Table 4).

Solid lipid nanocarriers (SLNs) and nanostructured lipid carriers (NLPs) have been tested for dermal and transdermal delivery, as well as regulatory aspects [7], and they have shown the following advantages compared to liposomes: higher drug loading, better drug stability, and prolonged release profile. Pectins have been tested for microencapsulation of different sensitive food components and are cost-effective [368].

The mathematical modeling includes empirical, semiempirical, and mechanistic models. The first two types of models are simple and practical, but they do not clearly elucidate the release mechanisms. The mechanistic models define the mechanisms of the release processes [363].

Some simplified models are developed and used to assess the controlled release of BAC. The zero-order kinetic models explain the prolonged release of the active agent/additive as core material and of encapsulated flavoring agents or sweeteners in products such as chewing gum. The zero-order kinetic results are accurate in the initial period of the release process. The Korsmeyer–Peppas and Ritger–Peppas models [369] are applied, considering three main assumptions: Short times were considered where M_t_/M_∞_ < 0.6, one-dimensional release; the ratio between the system length and thickness should be at least equal to 10. The Korsmeyer–Peppas equation is

(1) $$\frac{Mi}{M\infty }\;=\;k{\left(t\;-\;l\right)}^{n}$$

where M_∞_ and Mi are the concentrations of released (bio)active compound at equilibrium state and at time t, respectively, and l is the lag time. The power law equation is defined by the value of “n”, where n = 0.5 is the Fickian model (Case I) and the second one is (0.5 < n < 1), the non-Fickian model (Case II). Several model versions have been elaborated, such as the Peppas and Sahlin model, the Weibull model, the Hopfenberg model, the Cooney model, the Baker–Lonsdale model, the Hixson and Crowell model, etc. [363]. The selection of a model is frequently based on the correlation coefficient R (or the coefficient of determination, R^2^, which is the square of the correlation coefficient). A diffusion-controlled drug release from the developing gel layer has been found when this is very well hydrated and resistant to erosion. The erosion-controlled zero-order release is affected by the hydrodynamic stress except for some hydrocolloids with incomplete swelling. A new type of release mechanism is controlled by the polymer particle erosion.

(I) The mathematical definition of dissolution is based on the following equation:

*dC*/*dt* = *DA*/t (*Cs* − *C*) (2)

where *dC*/*dt*—the dissolution rate, D—the coefficient of diffusion, A—the solid surface area, Cs is the solubility, C is the concentration.

(II) For the diffusion mechanism, defined by the mass flux (J) as the mass amount (M) passing through a surface (S) during the time (t), expressed by Equation (3),

*J* = −*dM*/*dt*
*S* (3)

The steady state (concentration within the control volume does not change over the processing time) diffusion is described by Fick’s first law of diffusion. This law in one-dimensional geometry is as follows:

*J* = −*D* 𝜕*C*/𝜕*x* (4)

where J is the flux of diffusion (mol/m^2^/s), C is the concentration (mol/m^3^, position dependent), and D is the diffusion coefficient (m^2^/s). x represents the position. For unsteady state diffusion, Fick’s second law is applied, expressed by Equation (5),

𝜕*C*/𝜕*t* = −*D* 𝜕^2^*C*/𝜕*x*^2^ (5)

where C represents the position- and time-dependent concentration.

Non-Fickian modeling takes into account mass transport by the pressure gradient, dissolution, chemical degradation, etc. Fewer simplifications and assumptions for the prediction of the impact of food matrix microstructure, chemical composition, behavior under different conditions, and structure breakdown should be considered in the elaboration of the customized models. A sigmoid curve, with an inflection point corresponding to a change in the release rate, is specific to several release profiles. A dual-release model, recently developed, consists of the combination of a modified Korsmayer–Peppas model from the beginning to the inflection point and the Lumped model [370]. Applying this model, the maximum release rate, the dissolution efficiency, and other pharmaceutical parameters can be obtained, and they are useful to optimize the composition and the configuration of a drug delivery system. Establishing the parameters of the release kinetics can aid in reducing the dosage of some bioactive food ingredients (e.g., natural antimicrobials and antioxidants) that may cause an off-odor or off-flavor in food products when used in high amounts.

Mathematical models help to understand and predict the release behavior of BACs during transportation, storage, and applications and facilitate the development of new systems. The release modeling of (bio)active compounds from food to the gastrointestinal tract (GIT) in the living body through surface erosion is caused by the mechanical action of the stomach and diffusive mass transit. (acids and enzymes) As liquids diffuse from the GIT fluid to the food, food solutes (vitamins, fibers, oils, minerals, etc.) diffuse to the GIT medium with a concentration gradient. In many cases, Fick’s second rule of diffusion has been appropriate as the theoretical foundation to simulate the diffusion of GIT juice into various food systems [371]. Different kinds of mechanistic models have been proposed, such as the reservoir system (applied in lipophilic nutraceuticals), the matrix system (for antimicrobial food packaging systems in which no concentration gradient of bioactives is observed), and the swelling-based mechanistic models (for hydrogels). Parameters from mathematical models and the specific release mechanism have been considered to evaluate the quality and safety of foods/drugs [372]. Model-informed drug development (MIDD), as in silico computational, is known as an application of quantitative models in drug development to facilitate the decision-making process and application of exposure-based, biological, and statistical models derived from preclinical and clinical data sources [373].

## 7. Some Considerations on the Mechanisms of Antimicrobial, Antifungal and Antiviral Activities

### 7.1. Mechanism of the Antimicrobial Activity of the Bioactive Additives

The antimicrobial efficacy of essential oils is attributed to their ability to disrupt bacterial membrane integrity, to interfere with cellular respiration, and to induce oxidative stress, ultimately leading to microbial cell death. The versatility of EOs as natural antimicrobial agents has been demonstrated in their potential clinical and industrial applications as transdermal drug delivery and biologically active wound-healing compounds. Incorporating EOs into polymeric materials, especially those that regulate moisture and drug release, offers a promising and valid approach to optimize prevention and treatment strategies for infectious processes [126,374,375], offering a good solution for microbiological sanitization needs across various fields, particularly in environmental hygiene and food safety. Biomaterials of different structures, such as some polysaccharides (such as chitosan), proteins, peptides, etc., natural (bio)active compounds, and nanoscale materials, possess a broad spectrum of antibacterial/antimicrobial and antiviral activities that are explained by the different action modes depending on their characteristics (structure, size, shape, etc.) and the type of the microorganisms [376]. It is also well-known that the microorganisms can be classified based on their cell wall composition as Gram-positive or Gram-negative ones, by the nature of the cells (e.g., spores/vegetative), oxygen requirements (aerobic or anaerobic microorganisms), optimal temperature for growth (psychrophiles, mesophiles, thermophiles, or hyperthermophiles), etc. As it concerns the mechanisms of action, some AMs hinder essential metabolic or reproductive genetic pathways, whereas others alter the structure of microbial cell walls. Adhesion and biofilm formation by surface bacterial adhesion cause cross-contamination, food spoilage, and even diseases. The efficiency of natural antimicrobial agents and their applications could be improved by understanding the anti-adhesion mechanism [377].

**Figure 4:**
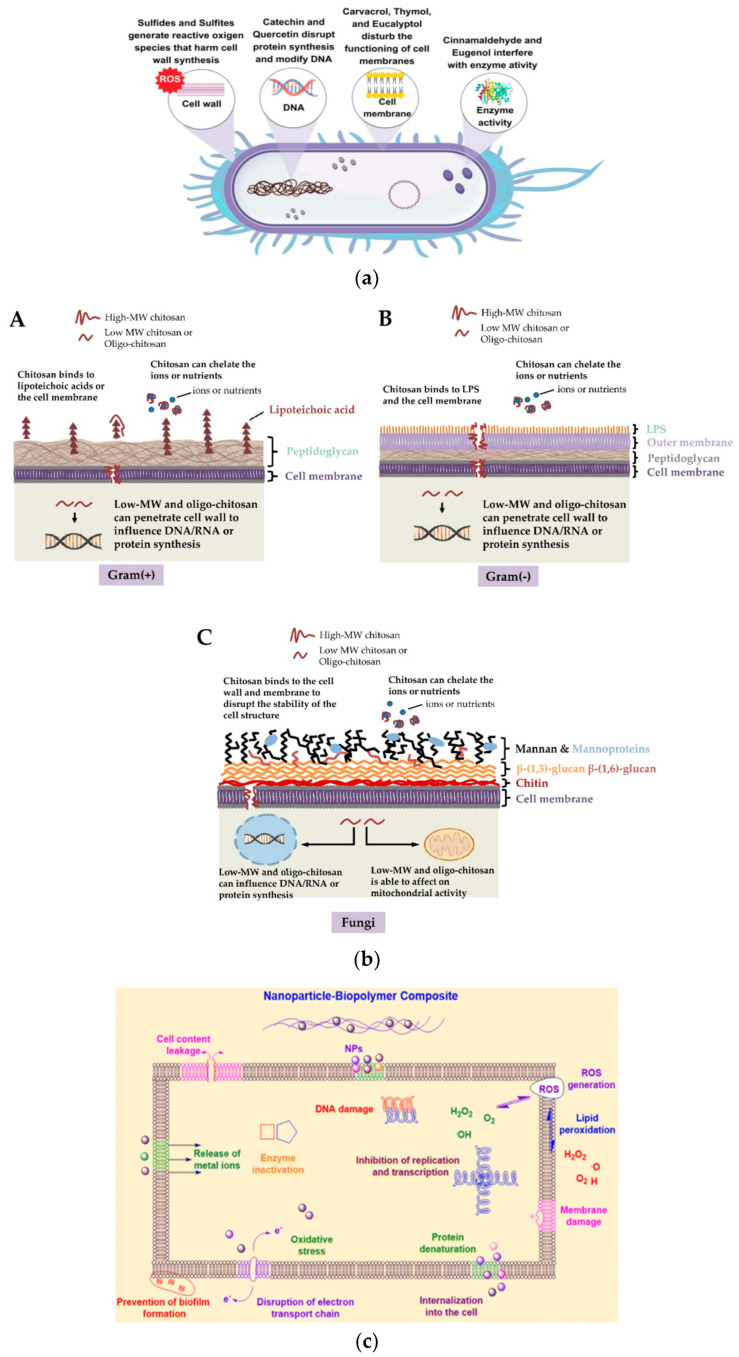
Mechanisms involved in antibacterial activity: (**a**) Mechanisms of action of plant antimicrobials against foodborne bacteria adapted from [380,381,382,383,384]. (**b**) Antimicrobial actions of chitosan against (**A**) Gram-positive bacteria, (**B**) Gram-negative bacteria, and (**C**) fungi [385]. (**c**) Antimicrobial mechanisms of action of NPs in nanocomposites designed for food packaging [282,336,386,387,388].

Mechanisms of action against foodborne bacteria of the antimicrobials from plants (polyphenols, EOs), animals (lysozyme, lactoperoxidase, lactoferrin), metabolites from microorganisms, or extracts are specific. The antibacterial agents, including natural preservatives, act by (Ι) bacterial cell membrane destruction/change in plasma membrane integrity; (ΙΙ) bacterial protein biosynthesis/essential metabolite synthesis; (ΙΙΙ) inhibition of bacterial cell-wall biosynthesis or disruption in protein synthesis; (ΙV) damage to bacterial DNA/RNA replication and repair/transcription; and (V) inhibition of a metabolic pathway or depolarization of the cell membrane [378,379]. Phytochemicals as biopreservatives exerted their potential antibacterial activities against multiple targets of sensitive and resistant pathogens via different mechanisms of action. The plant extracts modify the cell membrane of both Gram-positive and Gram-negative bacteria, which is evidenced by the potential disruption of the membrane of microorganisms, changes in cytoplasmic pH (pHint), and cell membrane hyperpolarization. Numerous new antimicrobial compounds have been isolated from endophytes as secondary metabolites, being promising sources for antimicrobials, particularly endophytic fungi and actinomycetes with substantial pharmacological potential [380]. Their actions include the direct physical contact mediated membrane disruption, which affects the nucleic acids, the decay of the proton motive force, and depletion of adenosine triphosphate (ATP)—Figure 4 [380,381,382,383,384,385].

Three main mechanisms of action for polyphenols have been proposed [381,382]: (i) the modification of the membrane permeability, (ii) the intracellular enzyme inactivation, and (iii) the modification of fungal morphology. Membrane structures of the Gram-positive and Gram-negative bacteria are different, exhibiting remarkable differences in their cell wall structure. For the Gram-negative bacteria, this comprises two membranes: the inner membrane and the outer membrane, separated by the periplasm. The outer membrane is an asymmetric bilayer, consisting of lipopolysaccharides and phospholipids, where many proteins are inserted. Transmembrane proteins form pores or channels called porin proteins, allowing the entry of hydrophilic molecules. The cell wall of Gram-positive bacteria is mostly composed of peptidoglycan. Teichoic acids associated with the peptidoglycan network reach the outer surface and are important antigens. They are polymers made up of units of glycerol phosphate or ribitol phosphate units or more complex units in which glycerol or ribitol is associated with sugars (glucose, galactose, etc.) and also contains D-alanine bound to glycerol or ribitol. Teichoic acids, as weakly anionic polymers, are found in the cell walls of Gram-positive bacteria. They can be classified based on their localization into wall teichoic acid and lipoteichoic acid. In the case of Gram-positive bacteria, electrostatic interactions are observed with lipoteichoic and teichoic acid, whereas Gram-negative bacteria contain lipopolysaccharides (LPS) in their outer membrane. Lipoteichoic acids are linked by covalent bonds to the membrane lipids through peptidoglycan. The outer membrane of the two bacteria generally has a negative charge. The mechanism of action of cationic antibacterial agents is on the outer membrane of the bacteria through electrostatic interactions. Bacteria are identified by coloring that varies on the membrane and their morphology. The coloring results from the fixing of the two dyes on the murein or peptidoglycan layer as a wall of polymeric nature. Gram-positive bacteria appear in purple in the presence of gentian violet, while the Gram-negative bacteria have a pink color in the presence of fuchsin. Antimicrobials and their binding modes also vary depending on their structure and bacterial species. Peptidopolysaccharides possess the ability to mimic the bacterial peptidoglycan layer and show antibacterial activity by interfering with bacterial cell wall synthesis. Antimicrobial peptides exert their bactericidal activities by inhibiting important intracellular processes like nucleic acid synthesis, cell wall synthesis, protein synthesis, etc. Finally, the compounds penetrate the bacterial membrane, altering its composition; reduce cell membrane potential, impairing vital cellular processes such as energy production, nutrient transport, and metabolic regulation; and the final result is the cell’s death [383]. The mechanisms by which polyphenols act against fungi are by blocking the efflux pump, affecting the cell membrane, interfering with ergosterol synthesis, damaging the cell wall, or generating reactive oxygen species (ROS).

The antimicrobial properties of chitosan and MIC depend on its structure, average molecular weight, degree of deacetylation, physico-chemical characteristics, and environmental conditions such as solvent type, pH, and the type of targeted microorganisms/fungi. Its effects against microbes can be extracellular, intracellular, or both, based on the targeting site and microorganisms. Chitosan with high molecular weight cannot penetrate the cell wall and cell membrane, but it acts as a chelator, preventing the extracellular removal of nutrients and altering cell permeability, while low-MW chitosan (<5 kDa) penetrates the cell wall and influences DNA/RNA or protein synthesis, intracellular antimicrobial activity, and mitochondrial function. Electrostatic interactions occur between the positively charged chitosan and the cell surface of the microorganism.

Biocidal activity of MOFs is explained by the activity of the metal ions released, which disrupts bacterial cell membrane integrity, and/or due to the active sites on the surface of MOFs, the protein and fatty acids present on the bacterial membrane have been degraded. In many cases, the synergistic effect in antimicrobial activity was observed by the participation of both metal ions (of Ag, Co, Zn, and Cu) and bioactive organic linkers. Gram-positive bacteria with a thick peptidoglycan layer penetrate with difficulty through the MOF’s surface. In the case of Gram-negative bacteria (i.e., bacteria with a thin peptidoglycan layer), a higher sensitivity has been observed. The use of MOFs for designing diagnosis techniques of the viruses, such as coronavirus, human immunodeficiency virus, and Ebola virus, has been tested [389].

### 7.2. Mechanism of the Antimicrobial Activity of NPs

All kinds of nanostructures initiate the process of antibacterial activity by direct physical contact with the bacterial membrane. The induction of membrane stress is followed by membrane damage. The process of generation of membrane stress differs among the different classes of nanostructures. Nanoparticles mostly adhere to the membrane surface and cause local perturbations, while nanotubes and nanosheets directly puncture and pierce the bacterial membranes using their sharp edges, corners, and narrow tips, creating pores in the lipid bilayer.

The antimicrobial process is initiated with bacteria contacting nanopatterns, which then impose mechanical stresses onto the bacterial cell wall, the activity being called “mechano-bactericidal” [386,390]. Antimicrobial agents migrate from nanoparticles to the microbial surfaces through simple molecular diffusion, micellar diffusion, or collisions. The antibacterial action of metal NPs includes, as a special step, the release of metal ions from the packaging system, the other steps being similar [387,388]. The main causes of the antibacterial action of NPs are (i) damage to the cell membrane by the electrostatic interactions of metal ions with the phospholipid bilayer of the bacterial membrane; (ii) oxidative stresses; (iii) generation of reactive oxygen species (ROS) as free radicals like the superoxide anion (O_2_^•−^), singlet oxygen, and the hydroxyl radical (OH^•^), lipid peroxides, which damage DNA and proteins; (iv) protein damage and loss of cell metabolism; and (v) damage to DNA and proteins that inhibits the enzymatic activities (Figure 4c) [388].

AgNPs can adhere to the cell surface and degrade lipopolysaccharides, forming a pit in the cell membrane. Antimicrobial activity of NPs depends upon a variety of factors, such as the physiological state of bacteria (planktonic or biofilms), their growth rate (stationary or starved), the ratio, the concentration of NPs and their characteristics, such as the type of material used for synthesis, size, shape (spherical, cubes, tubes, rods, and triangles), roughness, charge, chemical modification, and coating, environmental factors (pH, temperature, and aeration), and the physiological properties of NPs. The direct interaction of NPs must be with microbial cell surfaces by van der Waals forces, electrostatic forces, hydrophobic forces, and dominating receptor-ligand interactions, and also the penetration of the microbial cell membrane, interaction with cellular pathways, and altering the shape and function of the membrane. The metabolic pathway is also characterized by enzyme inactivation, protein denaturation, electrolyte imbalance, oxidative stress, and altered gene expression levels. The antimicrobial agents may move from the nanoparticles to the microbial surfaces through simple molecular diffusion, micellar diffusion, or collisions. The generation of oxidative stresses as ROS is observed for all types of nanostructures and leads to oxidative damage of lipids, proteins, nucleic acids, and membrane components. ROSs induce oxidative stress and the release of metal ions in solution. As an example, the antibacterial effect of AgNPs is mainly related to their adhesion to the bacterial wall, which interacts with cell membrane components (lipids and proteins), and subsequent penetration into cells, causing damage to DNA and proteins. The AgNPs can also interact with phosphorus and sulfur compounds within bacterial cells, thus affecting cell viability. The release of Ag^+^ ions increases the concentration of ROS inside the bacterial cell, leading to cell death after interaction with respiratory proteins.

## 8. Some Considerations on the Mechanism of Antioxidant Activity

Natural antioxidants are found in bio-sources predominant in natural extracts in plants, fungi, bacteria, algae, lichens, actinomycetes, edible vegetables, herbs, fruits, and spices, having a high content of phenolics, carotenoids, vitamins, and microelements. They are classified as exogenous and endogenous. Exogenous antioxidants originate from plants such as phenolic acids, flavonoids, stilbenes, carotenoids, lignans, coumarins, and vitamins. Endogenous antioxidants (ubiquinone, metallothioneins, albumin, uric acid, and glutathione) are produced in the body. Antioxidants inhibit free radical formation, reduce oxidative stress, improve immune function, and increase health longevity. Primary antioxidants prevent oxidation by scavenging ROS and reactive nitrogen species (RNS), while secondary antioxidants function by chelating different metal ions. Antioxidants prevent or delay oxidation at low concentrations, blocking UV light, radical scavenging, and chelation by hydrogen atom transfer (HAT) and electron transfer (ET or single electron transfer SET), and have the ability to chelate transition metals, depending mainly on their chemical structure. Phenolic compounds reduce or inhibit free radicals by transferring a hydrogen atom from their hydroxyl group. The position of OH and OCH_3_ groups on the phenolic rings predominantly affects the antioxidant potential of individual phenolic compounds. The antioxidant potential of a mixture can be additive, synergistic, or antagonistic [391,392]. In the agricultural and biological sciences area, antioxidant block the propagation stage in oxidative chain reactions of proteins, lipids, DNA, or other molecules [393]. Antioxidants scavenge free radicals and protect the body from stress, as it is a concerted transfer of the hydrogen cation from the phenol to the radical, forming a transition state [394]. Oxidative stresses affect macromolecules like proteins, lipids, and DNA, and subsequently cells/tissues. Gallic and caffeic acids have the ability to act by an HAT, while resveratrol has the ability to undergo an SET. Carotenoids, through three mechanisms— SET, HAT, and the formation of one adduct—protect cell membranes and lipoproteins against peroxyl radicals, being excellent peroxyl radical scavengers.

The reaction mechanisms of the antioxidant compounds are closely related to the reactivity and chemical structure of free radicals as well as the environment in which these reactive species are found. Free radicals in a biological system can be produced by exogenous factors, such as the presence of ultraviolet rays, which lead to the homolytic breakdown of bonds in molecules. The radical O_2_^•−^ does not react directly with polypeptides, sugars, or nucleic acids. The change in plant metabolic pathways is attributed to environmental stresses that result in ROS destroying membrane lipids, plant cells, DNA, and proteins by irreversible reactions. Antioxidants are important components in the signaling and defense mechanisms in some plants against pathogenic organisms and predators and are modulators of plant growth and the defensive system. Different plant antioxidants have the ability to counter reactive oxygen/nitrogen species. Antioxidants inhibit or quench free radical reactions mainly based on their reducing capacity or hydrogen atom-donating capacity, their solubility, and their chelating properties. The antioxidant reaction mechanisms of vitamin C are based on the HAT mechanism to peroxyl radicals, the inactivation of singlet oxygen, and the elimination of molecular oxygen. In general, HAT reactions are relatively independent of solvent and pH effects and are completed in a short time. HAT-based assays measure the capability of an antioxidant to quench free radicals (generally peroxyl radicals) by H-atom donation.

The HAT mechanisms of antioxidant action in which the hydrogen atom (H) of a phenol (Ar-OH) is transferred to an ROO• radical can be summarized by the reaction

ROO• + AH/ArOH → ROOH + A•/ArO•(6)

The SET mechanism of antioxidant action is based on the following reactions:

ROO• + AH/ArOH → ROO• + AH^+^/ArOH (7)

AH^+^/ArOH^+^ + H_2_O ↔ A•/ArO• + H_3_O^+^ (8)

ROO• + H_3_O^+^↔ ROOH + H_2_O (9)

These reactions are solvent and pH-dependent.

SET can be subdivided into single-electron transfer followed by a proton transfer (SET-PT) and sequential proton loss electron transfer (SPLET) [395]. Several different reactive oxygen species (ROS) are generated in vivo. They perform physiological functions but also have roles in certain human diseases, being able to reduce cardiovascular disease risks and some types of cancer, which in turn improves health and quality of life. ROS levels allow their physiological roles whilst minimizing the oxidative damage [396].

Lipid oxidation caused by the chain reaction of free radicals can be illustrated in three well-known stages: initiation, propagation, and termination [397].

Nanotechnology provides an improved absorption of antioxidants in human metabolic systems. Functionalized metal nanoparticles, transition metal oxides, and nanocomposites are potent nanoantioxidants. Many metabolically important compounds, like flavonoids, terpenoids, and oxidative stress-responsive agents, play a promising role in the capping and stabilization of the nanoparticles. Nanoantioxidants have several advantages over conventional antioxidants, such as increased bioavailability, controlled release, and targeted delivery to the site of action. Nanoceria, silica nanoparticles, polydopamine nanoparticles, polysaccharide-based and protein-based nanoantioxidants have been tested as biologically synthesized nanomaterials, which have emerged as significant alternatives due to their biocompatibility and high stability to increase/control their properties. The functionalization strategies are applied as gallic acid functionalization for improved scavenging, biofunctionalization of AgNPs, surface functionalization [398], or nanoencapsulation using lipids.

## 9. Toxicity Issues

Most of the active additives are not chemically bonded to the polymer matrix and can leach out during the life cycle of the materials, posing risks to human health and to the environment [399]. Natural preservatives have been recognized for their safety. However, these substances in large amounts can influence color, smell, and toxicity while being effective as food preservatives, having beneficial properties and having few side effects. Comprehensive toxicological studies are necessary to obtain the approval of plant antimicrobials as food preservatives from the European Food Safety Authority (EFSA), the Food and Drug Administration (FDA), and the China Food Additives Association (CFAA). Natamycin and nisin are currently the only natural preservatives being regulated, and other natural preservatives will have to be legally regulated before their widespread use. Such authorization must be based on comprehensive evaluations of factors such as purity, stability, toxicological studies, exposure and safety assessments, potential allergenicity, etc. Many plant antimicrobials have the GRAS status only for specific food applications, but their use in other applications is not expressly approved. Plant phenolics are actually absent in the positive list of food preservatives. Some essential oils can cause skin irritation and allergies in some cases. Some in vitro and in vivo studies have indicated that carvacrol, thymol and eugenol may have potential toxicological effects; after direct contact, they may act as allergens [400], such limitations being associated only with high concentrations. Thyme EO effects in the vapor phase may have implications for indoor air quality. Migration/controlled release of BC as drugs must be detailed and known to give the expected activity of these.

Another evidenced aspect is the interaction of polyphenolic food additives with the polymers in food matrices; such interactions could reduce antioxidant and antimicrobial effects and other biological properties, such as the availability, digestibility, and absorption of the macromolecules, the solubility of polyphenols in fatty food matrices, etc. Encapsulation is beneficial both for consumer health and the environment, as it could enhance the solubility and mask any unpleasant flavors, thereby broadening the application of phytoextracts from pomegranate and olives as food additives.

As it concerns the (poly)phenolic extracts, widely used for their antimicrobial/antioxidant and nutraceutical properties, the assessment of their life cycle (LCA) is necessary to understand their contribution to sustainability, starting with the natural resources and the emissions generated and the environmental impact, and to suggest the possible strategies to mitigate negative effects by means of water footprint and carbon footprint indicators [401]. Strategies that valorize by-products and reduce the use of chemical substances are essential for minimizing environmental pollution and addressing issues related to contamination. Extracts obtained from agro-industrial by-products have a significantly lower impact in terms of water pollution compared to pharmaceutical production and antibiotics/antimicrobials. Due to their potency, essential oils can be both environmentally sustainable and economically efficient.

Growing interest in NPs’ use in the food industry, cosmetics, and especially in biomedical applications has increased concerns about their safety and toxicity. It has been estimated that AgNPs production will increase from 360 to 450 tons annually to 800 tons by 2025. The AgNPs used in the food industry and for medical purposes may be limited due to high cost and toxic nature. The antimicrobial efficacy of AgNPs is significantly enhanced by their synergistic interaction with many well-known antibiotic drugs.

NPs are more chemically active and toxic than bulk materials due to the greater surface area and small size. NPs can potentially penetrate tissues, cells, and organelles and interact with functional biomolecular structures, causing toxicity. It is very necessary to design NPs with improved performance and reduced side effects. The mechanisms of toxicity/cytotoxicity and behaviors of the NPs are affected by various physico-chemical parameters, such as their nature, shape, structure, agglomeration state, surface charge, functionalization, wettability, dose, etc. Metal-based nanoparticles can harm the environment (e.g., soil, water) and have a negative impact on the life of organisms. Their use for biomedical purposes is severely limited by the inability to excrete or metabolize them in the human body. High doses of NPs can cause a disproportionate decrease in cell viability. NPs reach living bodies by inhalation, skin penetration, or ingestion, migration, or storage in packaging and nanosensors in landfills with the potential for release into the environment, air, water, and soil. In food applications, ingestion is the most obvious route for nanoparticles to come into contact with the human body. The gastrointestinal tract features, such as pH, the presence of various surface-active chemicals, electrolytes, digestive enzymes, and mechanical forces, alter nanomaterial absorption, potentially causing changes in nanoparticle properties and agglomeration state. The cytotoxicity of NPs depends on several factors, such as particle size, type of surface charge, concentration, and exposure/contact time of the packaging material with food. Upon uptake into the body, their size, morphology, surface charge, coating, and chemical composition are changed, and the response of biological systems to the materials and their toxicity are modified. Assay methods such as endotoxin and lactate dehydrogenase (LDH) signaling to apoptosis and oxidative stress detection supply valuable techniques for exposing biomarkers of nanoparticle-induced cellular damage. Spectroscopic investigation of epithelial barrier permeation and distribution within living cells reveals the NPs’ penetration, the natural defensive boundaries of the body, and their deposition in cytotoxic locations. A combination of the various characterization methodologies and assays is required for each situation of the investigated NPs. Novel methods suitable for the evaluation of nanoparticles are centered on nanoparticle toxicity analysis. The development of standardized methods for risk assessment and harmonized regulations to ensure safety by integrating organic nanomaterials into societal applications is necessary [402].

AgNPs have demonstrated potential anticancer effects against human colon cancer (HCT-116) cells [403]. ZnO NPs showed cytotoxicity against two human intestinal cell lines (Caco-2 and LT97—human colon adenoma cell line). The NPs containing positive surface charges have a more significant harmful effect on cell membranes, causing cell death. Microwave cooking promoted AgNPs migration more than a regular oven. Although metal and metal oxide NPs are frequently referred to as biocompatible materials, their migration of NPs is negligible, and they have no notable harmful effects in vivo or in vitro. Proinflammatory reactions and oxidative stresses are present, so a food safety concern exists in the mind of the consumer. Low concentration of AgNPs and TiO_2_ NPs migration from the packaging to the cell explains the low toxicological potential of the released metal ions from packaging, insufficient to cause harm to human cells. Engineered nanomaterials presented in food must be outlined in the list of ingredients and nanomaterials used in food contact materials, which must be explicitly authorized, and a specific risk assessment of the nanomaterial must be carried out. There is also a need to assess the toxicity of nanomaterials, including chronic exposure and carcinogenicity, and the presence of AgNPs in water bodies has emerged as a new environmental concern. The efficient separation of these nanoparticles remains a critical challenge. Cytotoxic effects of NPs on normal cells and living organs hinder their use in clinical trials. Toxic effects of NPs should be elucidated after studies on transport, accumulation, degradation, and elimination using both in vitro and in vivo models on various organs to reveal their efficacy and impact on health [404].

## 10. Concluding Remarks and Future Trends

The natural (bio)active compounds extracted from various resources, including new species of plants, animals, microorganisms, algae, marine by-products, or biowaste, and by using novel advanced extraction techniques, have different compositions and functions. A detailed characterization of active additives evidenced new aspects related to their composition dependence on plant type and cultivation conditions, extraction and further processing by emulsion/encapsulation, drying, storage, etc. Active additives are widely recognized for their significant contribution to enhancing the overall quality and stability of various products. They are very interesting and have great potential for the market of functional materials, especially when they have been extracted under mild conditions for better retention of their activities/functionalities.

Drying is a vital postharvest process for plants to decrease their moisture content, to retain the organoleptic characteristics, and to prevent oxidation and enzymatic breakdown and to inhibit microbial growth in the dried plants. It is one of the oldest and most effective methods for preserving medicinal plants, consequently preserving the valuable compounds and extending their shelf life. The BACs as components of natural additives are easily degraded by thermal treatment, pH, oxidation, light, and/or hydrolysis, and other actions, including enzymes. The agricultural products and foods that have been heated for a long time lose their bioactive compounds and their qualities. During heating, their hydroxyl groups are often oxidized to aldehyde, ketone groups, or carboxyl groups. The effects of the drying process on the major bioactive/effective compounds are very pronounced and undesirable. It has been established that vacuum drying and vacuum freeze drying are better than other methods applied to keep the quality/activities of the natural actives. For example, the ideal drying temperatures for retaining qualities of the vitamin C, polyphenols, flavonoids, glycosides, volatile compounds, and their antioxidant activity should be 50–70 °C [405]. The changes occurring in BAC as vitamins, polyphenols, flavonoids, glycosides, and volatile compounds in fruits, vegetables, spices, and medicinal and aromatic plants, as well as their antioxidant activity, must be established before practical applications. These effects should be studied in detail to be avoided.

The complexity of plant antimicrobial mixtures and the need for extensive safety data create challenges and barriers in the regulatory approval process to obtain GRAS status, which is a time-consuming and costly approval process.

The green extraction techniques are required to be evaluated in terms of their safety, scalability, consumer acceptability, challenges, legal aspects, and potential feasibility. The lack of in vitro/in vivo/clinical research on essential oils is one of the limiting factors for their application. Recently, the development of an in vitro cultivation technology coupled with a study of the biochemical and antimicrobial properties of the EOs with reference to those of *Salvia rosmarinus* [406] has been proposed. Well-designed studies on animals and humans are necessary to assess the efficacy and safety of natural active additives and NPs, and it is recommended to conduct detailed toxicological assessments for each kind of additive selected for an intended associated application.

Some compounds in EOs can be toxic and cause side effects. Many volatile components in essential oils, due to their mixtures, are difficult to standardize for the application amount, as are the EO preservatives with optimal in vivo effects in food preservation. There are a few experiments evaluating the safety of EOs in humans, but. These indicators depend on the applied extraction method (hydroethanolic extraction shows higher CO_2_ emissions than aqueous extraction). Consequently, further studies on sensory analysis and consumer acceptance are necessary. The translation of in vitro studies to in vivo experiments and finally to human clinical trials has been the major challenge in the development of new phytochemicals and other natural additives, and also new synthesized inorganic and biopolymer NPs. The phytochemicals play an important role in bio-reduction processes, and NPs have a selective toxicity for the diseased tissues. Green synthesis of NPs using phytochemicals solved some problems related to their toxicity. However, it is still needed to assess the toxicity of nanomaterials, including chronic exposure and carcinogenicity. Antimicrobial packaging is an important alternative to active packaging, prolonging shelf life and ensuring food safety. Despite these benefits, the antimicrobial packaging containing eco-friendly materials and having adequate mechanical and barrier properties is still a difficult task for both industries and consumers. A promising solution for producing antimicrobial food packaging with enhanced mechanical properties consists of the integration of nanostructures or NPs in active packaging.

Biodegradability is crucial for environmental sustainability, as it enables the material to decompose naturally in environments, reducing waste accumulation. Natural degradation is hindered by the hydrophobic properties of the polymers and stability/stabilization by the incorporation of suitable additives. Polyphenolic compounds affect the long-term degradation behavior of polymer and composite materials. The effect of the incorporation of polyphenols is a significant improvement in biological functionality and some of the physico-chemical properties of synthetic polymers. PLA is one of the most prevalent biodegradable plastics, with extensive applications in packaging, transportation, and medical fields. The PLA biodegradation has been analyzed using specific enzymes such as proteinase K, PLA depolymerase, and lipase. It has been established that in natural environments, enzymes synergistically work by an efficient sequential catalytic process. Microbial degradation is a safe and highly effective method for the degradation of polymer materials containing AC.

Two aspects have to be mentioned: The surprise of the research/use of the natural products is that they still offer new important aspects, revealed in recent years, concerning their composition and properties/activities relationships, even if they have been studied for centuries, and active nanoscale-sized inorganic and organic additives, which separately or in combination with natural active additives, help to find innovative solutions for current needs of society in many fields. This is reflected by an impressive number of references in a short period (as in 2023–2025 years—cited refs of this review). Therefore, we decided to split the review into two parts; in the second one, the applications (in agriculture, food, biomedicine, cosmetics, etc.) of both types of active additives will be presented. Active, bioactive, or functional additives are used in different applications to enhance product performance. For food and beverages, they improve shelf stability, taste, texture, appearance, and nutritional value. While in the pharmaceutical industry, they modify the drug delivery/release and efficacy, and they also impart specific properties like antimicrobial action, antioxidant protection, water and barrier properties, or UV resistance. Known applications are also in cosmetics, textiles, and other industries.

This review intends to serve as a guide for those interested in the future development of innovative materials with multipurpose applications by green procedures and as a novelty to the scientific community.

## Data Availability

No new data were created or analyzed in this study. Data sharing is not applicable to this article.
